# Unusual fcc-structured Ag_10_ kernels trapped in Ag_70_ nanoclusters†
†Electronic supplementary information (ESI) available: IR, ^13^C NMR, CV, UV, EDS, PXRD and luminescence decay curve, and details of the data collection and structure refinements, and crystal data. CCDC 1850394 and 1850395 for **SD/Ag80a** and **SD/Ag80b**. For ESI and crystallographic data in CIF or other electronic format see DOI: 10.1039/c8sc03396j


**DOI:** 10.1039/c8sc03396j

**Published:** 2018-10-18

**Authors:** Yan-Min Su, Zhi Wang, Gui-Lin Zhuang, Quan-Qin Zhao, Xing-Po Wang, Chen-Ho Tung, Di Sun

**Affiliations:** a Key Lab for Colloid and Interface Chemistry of Education Ministry , School of Chemistry and Chemical Engineering , Shandong University , Jinan , 250100 , People's Republic of China . Email: dsun@sdu.edu.cn; b College of Chemical Engineering and Materials Science , Zhejiang University of Technology , Hangzhou , 310032 , People's Republic of China

## Abstract

Controlled trapping atom-precise ultrasmall silver nanoparticles into silver nanoclusters is challenging; thus only limited progress has been made in this area. We are therefore inspired to isolate two novel silver nanoclusters, Ag_10_@Ag_70_ (**SD/Ag80a** and **SD/Ag80b**; SD = SunDi), where a novel fcc-structured Ag_10_ kernel built from two single-edge opened Ag_6_ octahedra by sharing one edge is trapped. The bioctahedral Ag_10_ kernel is locked by a pair of Mo_7_O_26_^10–^ anions to form an inner Ag_10_@(Mo_7_O_26_)_2_ core which is further encapsulated by an outer Ag_70_ shell to form three-shell Ag_10_@(Mo_7_O_26_)_2_@Ag_70_ nanoclusters. Notably, the bioctahedral Ag_10_ kernel has not been observed in silver nanoclusters ever before, thus representing a new embryo state of silver nanoparticles. **SD/Ag80a** emits in the near infrared (NIR) region (*λ*_em_ = 730 nm) at low temperature. This work will deepen our understanding on the atomic-level growth of silver nanoparticles and complicated three-shell self-assembly involving polyoxometalate (POM) and two different silver nanoclusters.

## Abstract 2

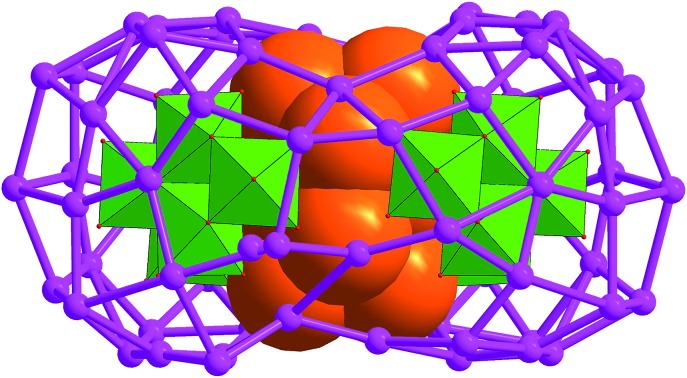
A bioctahedral Ag_10_ kernel is locked by a pair of Mo_7_O_26_^10–^ anions to form an inner Ag_10_@(Mo_7_O_26_)_2_ core which is further encapsulated by an outer Ag_70_ shell to form three-shell Ag_10_@(Mo_7_O_26_)_2_@Ag_70_ nanoclusters.

## Introduction

Ultrasmall silver nanoparticles (*e.g.*, few-atom clusters, <1 nm) represent the embryo states of larger silver nanoparticles (typically >2 nm) to some extent, which have defined molecular structures and compositions and thus can deepen the understanding on the size evolution of silver nanoparticles.^1^ Given this, X-ray single crystal structures become a prerequisite to get atomic-level information including the surface ligands, inorganic–organic interfaces and silver atoms packed in silver nanoparticles.^2^ While chasing large silver nanoclusters such as Ag_14_, Ag_21_, Ag_23_, Ag_44_, Ag_50_, Ag_62_, Ag_67_, Ag_74_, and Ag_141_ and even the largest known Ag_374_,^3^ chemists almost neglect the significance of the embryo states of silver nanoparticles that however are quite difficult to be captured due to their typical kinetics-controlled growth course.^4^ Therefore, controlling the reductive transformation from Ag(i) to Ag^0^ and then trapping the transient Ag aggregates into the thermodynamically stable crystalline product during the self-assembly is an urgent need and thus a major challenge.

Learning from the solvent-controlled synthesis of multiple-twin decahedral and icosahedral silver nanoparticles with special favourable [111] facets,^5^ we found that DMF (*N*,*N*-dimethylformamide), compared to widely used NaBH_4_, is a much more mild reductive agent which facilitates the formation of Ag_6_ octahedral kernels during the slow reduction process as seen in Ag_34_ and Ag_62_ nanoclusters.^6^ Such Ag_6_ octahedra can be seen as the smallest fragment cut from the unit cell of face-centered cubic (fcc) bulk silver metal, whereas other silver nanoclusters smaller than the most common icosahedral Ag_13_ are still not directly observed in any reported silver nanoclusters.^7^ Thus, the species in the early evolution from discrete Ag atoms to the metallic state are still largely vague and the exploration of a suitable synthesis strategy to trap them is scientifically desired.

With these considerations in mind, we used a DMF-containing mixed solvent system to isolate two novel silver nanoclusters [Ag_10_@(Mo_7_O_26_)_2_@Ag_70_(MoO_4_)_2_(CyhS)_36_(CF_3_SO_3_)_16_(DMF)_6_]·2DMF·4^*n*^PrOH (**SD/Ag80a**; SD = SunDi; CyhSH = cyclohexanethiol) and [Ag_10_@(Mo_7_O_26_)_2_@Ag_70_(MoO_4_)_2_(^i^PrS)_36_(CF_3_SO_3_)_16_(DMF)_6_] (**SD/Ag80b**). Two silver nanoclusters have the same metallic core but different organic coatings. In the innermost of cluster, an unusual fcc-structured Ag_10_ nanocluster constructed from two single-edge opened Ag_6_ octahedra by sharing one edge is locked by a pair of Mo_7_O_26_^10–^ anions to form an inner Ag_10_@(Mo_7_O_26_)_2_ core which acts as a template to support the outer Ag_70_ nanocluster to form a final three-shell Ag_10_@(Mo_7_O_26_)_2_@Ag_70_ nanocluster. This unprecedented bioctahedral Ag_10_ nanocrystal can be deemed as a new nanofragment cut from fcc silver metal and represents a possible transient species in the growth of large silver nanoparticles.

## Results and discussion

### X-ray structures of **SD/Ag80a** and **SD/Ag80b**

**Scheme 1:**
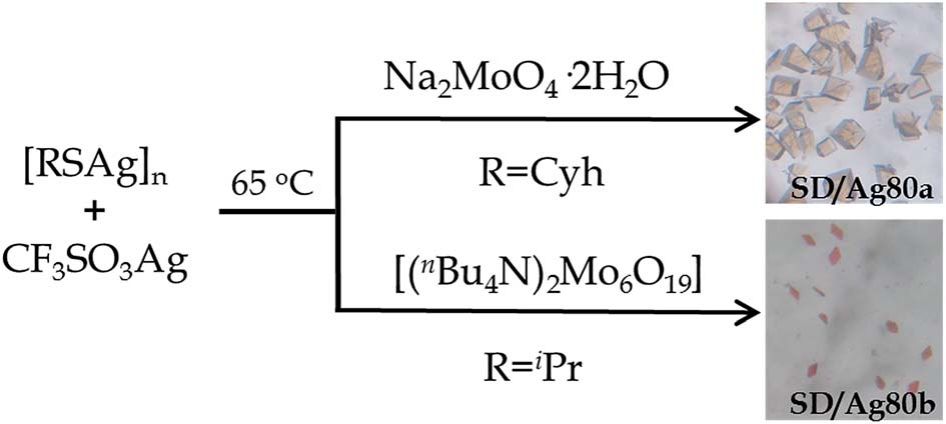
Synthetic routes for **SD/Ag80a** and **SD/Ag80b**.

**SD/Ag80a** and **SD/Ag80b** were synthesized through a facile one-pot solvothermal reaction of silver-thiolate polymeric precursors, CF_3_SO_3_Ag and molybdates in different DMF-containing mixed solvent systems (Scheme 1). In spite of several attempts, we still couldn't isolate **SD/Ag80a** and **SD/Ag80b** using the same Mo sources. Their samples were collected as brown-yellow and red crystals, respectively, after evaporation of solvents at room temperature for 1–2 weeks. Several synthetic parameters were optimized and are listed in Tables S1 and S2 (ESI)† for details. Details of the synthesis and some basic characterization are shown in the ESI.†

The molecular structures of **SD/Ag80a** and **SD/Ag80b** were revealed by single-crystal X-ray diffraction (SCXRD) analysis. They crystallize in monoclinic *P*2_1_/*n* and triclinic *P*1[combining macron] space groups, respectively. In each asymmetric unit only half of the corresponding clusters were resolved. Due to the structural similarities, only that of **SD/Ag80a** is described in detail here. The structural diagrams of **SD/Ag80b** are shown in Fig. S1.† Selected details of the data collection and structure refinements are listed in Table S3.†

**Fig. 1:**
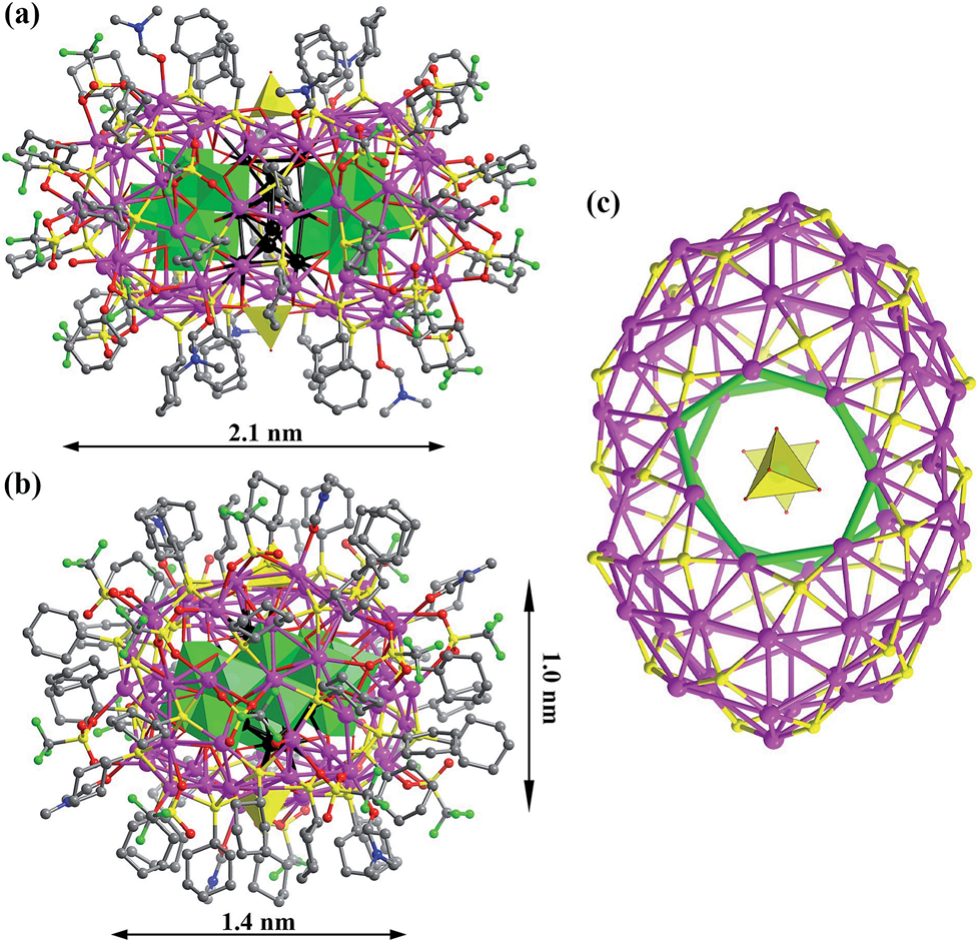
(a) and (b) The X-ray crystal structure of Ag_10_@(Mo_7_O_26_)_2_@Ag_70_ nanoclusters viewed along two orthogonal directions. The inner silver atoms of the Ag_10_ kernel are highlighted in black. Mo_7_O_26_^10–^ and MoO_4_^2–^ are represented by green and yellow polyhedra, respectively. (c) The Ag_70_S_36_ shell with silver heptagons highlighted in green.

**SD/Ag80a** is an elongated spheroid (1.0 × 1.4 × 2.1 nm) that sits on the crystallographic inversion center (*i*). The Ag_80_ nanocluster is composed of a Ag_70_ shell and a Ag_10_ kernel. The Ag_70_ shell is capped by 36 CyhS^–^, 16 CF_3_SO_3_^–^, 2 MoO_4_^2–^ and 6 DMF (Fig. 1a and b). All cyclohexyl groups of 36 CyhS^–^ ligands show a unified chair configuration. Two different coordination modes (μ_3_ and μ_4_) are found in 36 CyhS^–^ ligands capped on the silver trigons or tetragons (Ag–S distances: 2.389(5)–2.722(5) Å). The 16 CF_3_SO_3_^–^ anions exhibit three different coordination fashions including μ_3_-η^1^:η^2^:η^0^, μ_3_-η^1^:η^1^:η^1^, and μ_2_-η^1^:η^1^:η^0^. Two MoO_4_^2–^ anions (yellow tetrahedra in Fig. 1) adopt a μ_8_-η^2^:η^3^:η^3^ mode to bind in the equatorial region of the Ag_70_ shell. Six DMF molecules as terminal ligands finished the organic ligand coverage on the surface of the Ag_70_ shell. Three different O donor ligands (CF_3_SO_3_^–^, MoO_4_^2–^, and DMF) interact with Ag atoms with the bonding distances in the ranges of 2.406(15)–2.789(17), 2.251(11)–2.568(11) and 2.390(13)–2.458(14) Å, respectively. The Ag_70_ shell was further consolidated by the argentophilic interaction^8^ ranging from 2.833(2) to 3.4394(16) Å. The surface of the Ag_70_ shell consists silver trigons, tetragons, pentagons and heptagons (Fig. 1c). The silver trigons, tetragons, and pentagons are capped by CyhS^–^ or CF_3_SO_3_^–^, whereas MoO_4_^2–^ shapes the large silver heptagons (green rings in Fig. 1c).

**Fig. 2:**
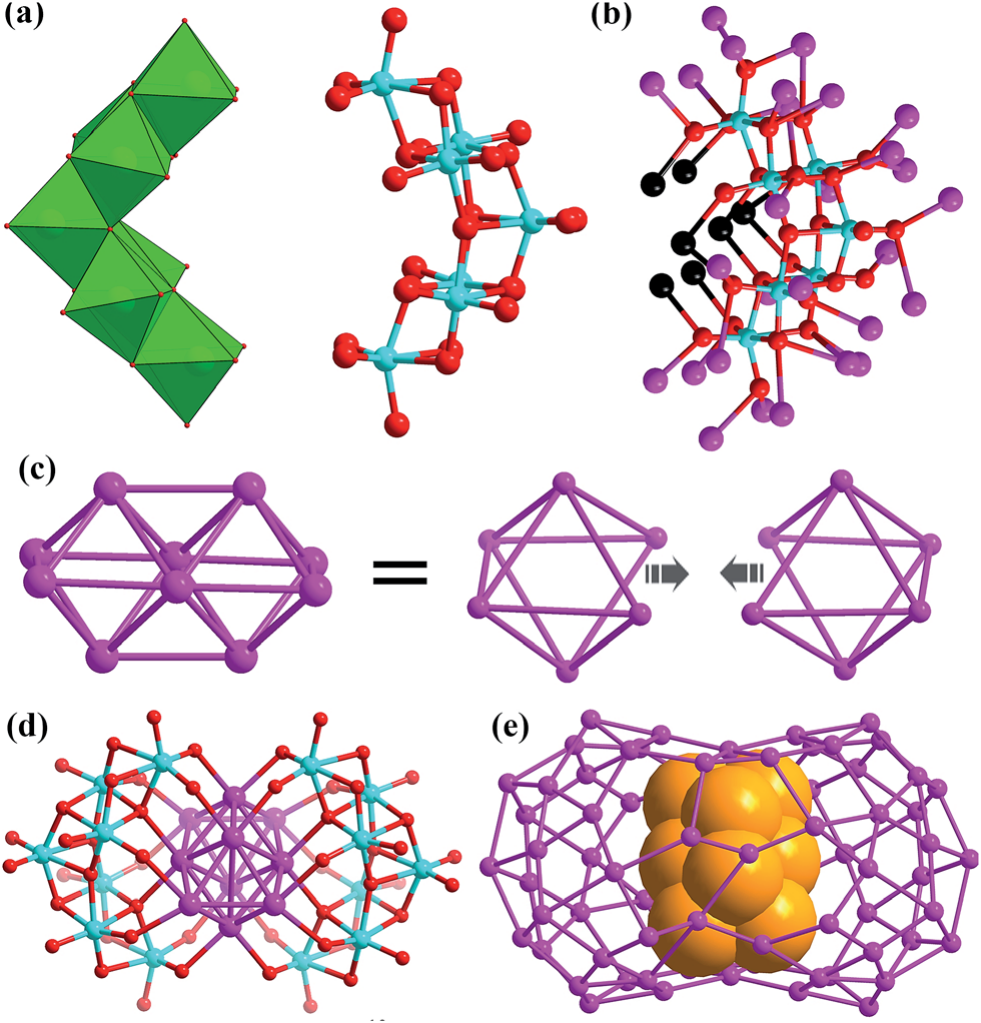
(a) Two Mo_7_O_26_^10–^ anions in **SD/Ag80a** shown in polyhedral (left) and ball-and-stick modes (right). (b) Binding fashion of Mo_7_O_26_^10–^ toward 35 Ag atoms (black: Ag from the Ag_10_ kernel; purple: Ag from the Ag_70_ shell). (c) Animation showing the formation of a Ag_10_ bioctahedron from two single-edge opened Ag_6_ octahedra by fusing one Ag–Ag edge. (d) The Ag_10_ bioctahedron locked by a pair of Mo_7_O_26_^10–^ anions. (e) The Ag_10_ bioctahedron (claybank space-filling balls) residing in the Ag_70_ shell.

There are two crescent-like Mo_7_O_26_^10–^ anions under the Ag_70_ shell (Fig. 2a). During the synthesis of **SD/Ag80a** and **SD/Ag80b**, although different Mo sources, Na_2_MoO_4_·2H_2_O and [(^*n*^Bu_4_N)_2_Mo_6_O_19_], were used, respectively, the same Mo_7_O_26_^10–^ anion was trapped as the template in the final silver nanoclusters. Thus, the novel Mo_7_O_26_^10–^ anions should be *in situ* transformed from Na_2_MoO_4_·2H_2_O or [(^*n*^Bu_4_N)_2_(Mo_6_O_19_)] in different solvent environments. We used Bond-Valence Sum (BVS) calculations for seven Mo atoms of Mo_7_O_26_^10–^, which confirmed that all of them are in the +6 oxidation state (Table S5†).^9^ The Mo_7_O_26_^10–^ is constructed from seven edge-shared MoO_6_ octahedra. The total 26 O atoms are divided into four kinds based on their binding fashion to Ag atoms, 2 μ_0_, 4 μ_1_, 16 μ_2_, and 4 μ_3_. Such highly negative-charged Mo_7_O_26_^10–^ totally binds 35 silver atoms. Among them, 7 are from the inner Ag_10_ kernel and the remaining 28 are from the Ag_70_ shell (Fig. 2b). Notably, this novel molybdate has neither been observed in classic POM chemistry nor in silver nanoclusters. More importantly, this molybdate carries the second highest negative charges^10^ which effectively enhanced its template effect by binding more Ag atoms (Table S6†).

The most interesting feature in **SD/Ag80a** is the unusual Ag_10_ kernel underlying the equatorial region of the Ag_70_ shell which is built from two single-edge opened Ag_6_ octahedra by sharing one edge (Fig. 2c). The shared edge is the longest Ag···Ag edge (Ag38···Ag38^*i*^ = 3.457(1) Å, symmetry code *i*: –*x* + 1, –*y* + 1, –*z* + 1) within the Ag_10_ kernel, which is out of the normal Ag···Ag interaction range. All other eleven Ag···Ag edges are distributed in the range of 2.659(2)–2.980(1) Å (Fig. S2†) and the average Ag···Ag distance is 2.814 Å, which is 2.5% shorter than the Ag···Ag distance in metallic silver (2.886 Å),^11^ indicating strong argentophilic interactions as in bulk silver metal. All exposed trigons of the Ag_10_ bioctahedral kernel are [111] facets which are capped by Mo_7_O_26_^10–^ anions through Ag–O bonding (Ag–O distances: 2.284(10)–2.433(10) Å; Fig. 2d). As such, the Ag_10_ bioctahedron is doubly clamped by a pair of Mo_7_O_26_^10–^ anions to form an inner Ag_10_@(Mo_7_O_26_)_2_ core, which was enwrapped by an outer Ag_70_ shell to form a three-shell Ag_10_@(Mo_7_O_26_)_2_@Ag_70_ nanocluster. The two polar sites of the Ag_10_ bioctahedron are also linked with the outer Ag_70_ shell through argentophilic interactions (Ag···Ag: 2.8523(19)–3.3621(18) Å; Fig. S3†).

Although the single Ag_6_ octahedron has been observed in several inorganic compounds^12^ and a few silver nanoclusters,^13^ its dimer, Ag_10_ bioctahedron, has never been observed before in silver nanoclusters. Such a Ag_10_ bioctahedron can be seen as a bigger nanofragment than a Ag_6_ octahedron. An important driven force for its formation should be the suitable reducibility of DMF.^6^ The oxidation product of DMF in the assembly process is Me_2_NCOOH^14^ which can be recognized from the ^13^C NMR (nuclear magnetic resonance) of HCl digested reaction mother solution (Fig. S4†). In the chemical shift scale corresponding to aldehydes and carboxylates (*δ* = 150–200 ppm), two peaks appeared at *δ* = 164.64 and 162.92 ppm, which are assigned to the carbon resonances of DMF and Me_2_NCOOH, respectively. We didn't observe any peaks in ^13^C NMR corresponding to the oxidation product of ^*n*^PrOH, which clearly excluded the possible reductive effect of ^*n*^PrOH in this assembly system. These results clearly evidenced the redox reaction between Ag(i) and DMF occurred during the self-assembly process. The emergence of a fcc-structured Ag_10_ nanocluster, on the other hand, answered an important question, which is how the common observed smaller Ag_6_ kernel grew up to larger structures. Based on the above structural information, we can tentatively assign a new edge-fusion mode to its growth mechanism, although several other growth modes for noble metal nanoparticles have been proposed such as face-fusion, interpenetration, shell-by-shell, layer-by-layer, and tetrahedron-based vertex-sharing growth modes.^2^ Based on the formulae and charge neutrality considerations, we can determine that the valence of the Ag_10_ kernel is +6, which means such a kernel carries four free electrons, belonging to a 4e superatom network. We also performed DFT calculations at the B3LYP/SDD theoretical level to study the free electron distributions on the frontier orbitals of the Ag_10_ kernel (see details in the ESI†). According to the results identified experimentally, the inner Ag_10_ kernel features *C*_i_ symmetry with +6 valence and four free electrons. Thus, frontier molecular orbital analysis (Fig. S5†) reveals that four free electrons occupy two *A*_u_-symmetry HOMO-1 and HOMO. HOMO-1 and HOMO exhibit different components. HOMO involves in the 5s orbitals of two ends of Ag_10_, while HOMO-1 concentrates on the 4d orbitals of two ends of Ag_10_. Moreover, HOMO-2 features *A*_g_ symmetry with similar components to HOMO-1, and LUMO consists of 5s orbitals in the centre of Ag_10_.

Combining the structural analysis and DMF-involved reductive process, we tentatively proposed a total shell-by-shell formation mechanism for such new silver nanoclusters. Weakly reductive DMF firstly induced the formation of an inner Ag_10_ kernel (1st shell), which exposes highly active [111] facets that are quickly passivated by the formation of Ag–O interaction with Mo_7_O_26_^10–^ (2nd shell). The inner [Ag_10_@(Mo_7_O_26_)_2_] core acts as the authentic template to support an outer Ag_70_ shell, forming the final core–shell type silver nanoclusters. Such a formation route resembled the mechanism revealed in the [Ag_6_@(MoO_4_)_7_@Ag_56_] family by electrospray ionization mass spectrometry.6*b*

We also noted that the Au_21_(S-Adm)_15_ nanocluster has been reported by the Zhu group,^15^ who firstly found the bioctahedral Au_10_ kernel formed by edge-sharing of two single-edge opened Au_6_ octahedra. However, the shared edge is not the longest one (opened edge) and the overall Au_6_ octahedral framework is severely disordered. Anyhow, as a counterpart of this Au_10_ kernel, the bioctahedral Ag_10_ kernel has not been reported before in silver nanoclusters.

### The optical properties of **SD/Ag80a**

**Fig. 3:**
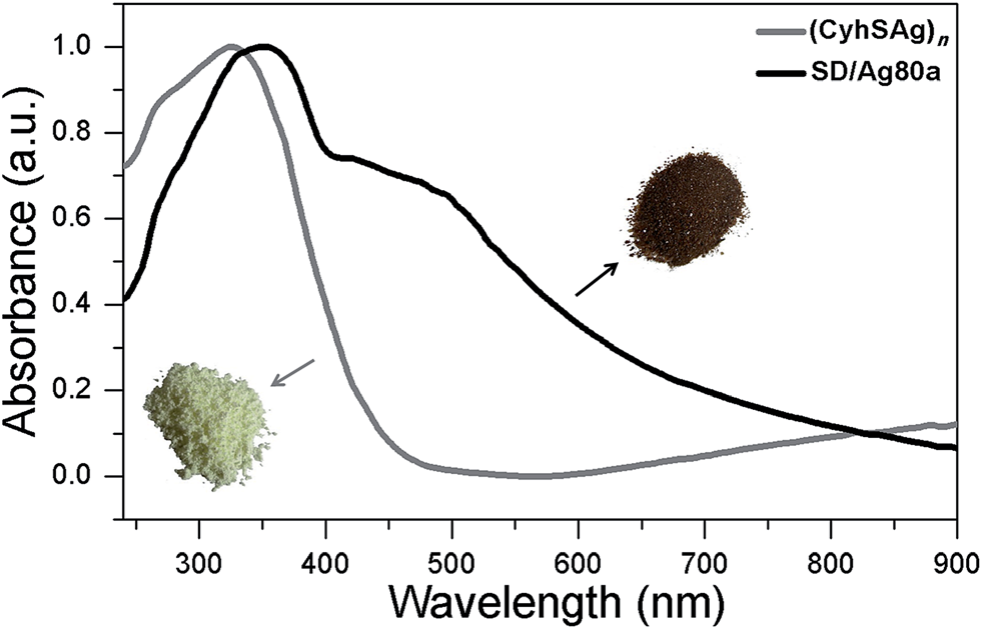
Optical absorption spectra of **SD/Ag80a** and the silver-thiolate precursor. Insets are photographs of solid samples of **SD/Ag80a** (brown microcrystals) and the polymeric precursor (CyhSAg)_*n*_ (pale yellow powder).

The UV/Vis spectrum of **SD/Ag80a** was measured in the solid state using diffuse reflectance mode. As shown in Fig. 3, **SD/Ag80a** showed an absorption maximum at 344 nm and a shoulder peak in the visible region (∼490 nm), which should be ascribed to ligand-based absorption and the charge transfer transition from the S 3p to Ag 5s orbitals, respectively. Similar assignments were also made in a hypothetical silver sulfide monomer and molecular [Ag_62_S_13_(SBu^*t*^)_32_]^4+^ cluster.^16^ Based on the Kubelka–Munk function (Fig. S6†), the band gap of **SD/Ag80a** was estimated to be ∼1.06 eV, which indicates that **SD/Ag80a** is a potential narrow-band-gap semiconductor. In comparison, the optical energy gap of the precursor (CyhSAg)_*n*_ is ∼2.09 eV.

**Fig. 4:**
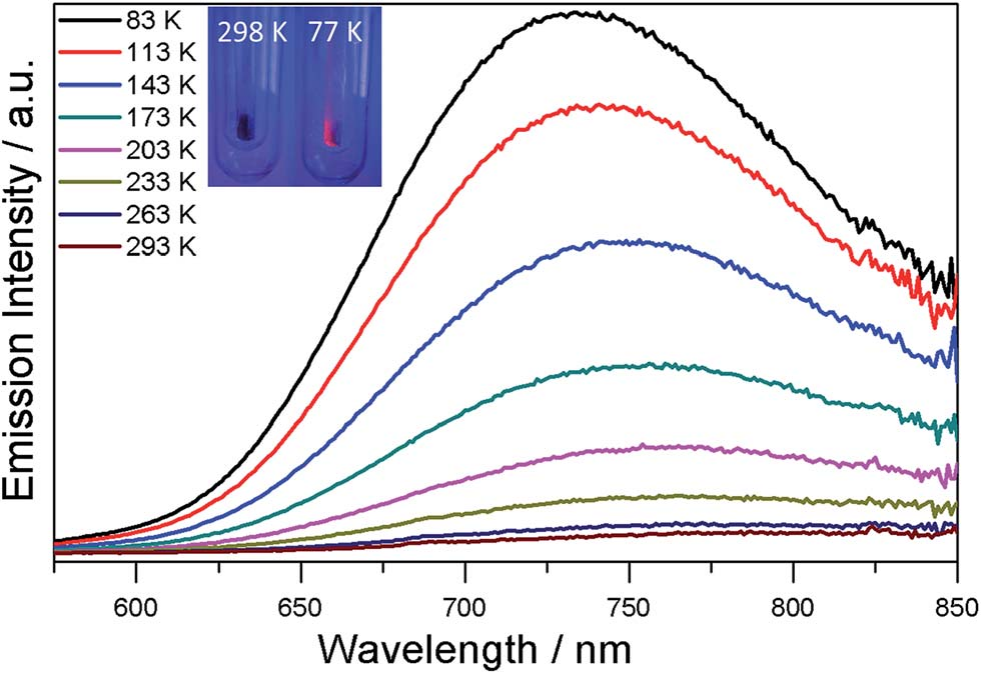
Varied-temperature luminescence spectra of **SD/Ag80a** from 293–83 K in the solid state. Insets show the photographs of the sample **SD/Ag80a** under a hand-held UV lamp (365 nm) at 298 and 77 K.

The luminescence properties of **SD/Ag80a** were studied in the solid state. As shown in the insets of Fig. 4, we can observe that **SD/Ag80a** isn't emissive under the UV light irradiation (*λ*_ex_ = 365 nm) at room temperature; however, it emits red luminescence at 77 K. The varied-temperature emission spectra of **SD/Ag80a** in the solid state were recorded from 293 to 83 K with 30 K as an interval, showing luminescence thermochromic behavior. When gradually cooled to 83 K, the intensity of emission shows an 18-fold enhancement, which should be assigned to the low-temperature induced increase of radiative decay. The emission maximum was blue-shifted from 754 to 730 nm (*λ*_ex_ = 469 nm) in the temperature range of 173–83 K (Fig. 4), which may be related to the enhanced molecular rigidity at lower temperature.^17^ This near-infrared (NIR) emission should be assigned to ligand-to-metal-charge-transfer (LMCT) transition from S 3p to Ag 5s orbitals.^18^ The emission lifetime of **SD/Ag80a**, falling on the microsecond scale at 83 K (Fig. S7†), suggests the triplet phosphorescence origin.

## Conclusions

In conclusion, we developed a DMF-controlled strategy to successfully capture an atom-precise ultrasmall Ag_10_ kernel into a gigantic silver nanocluster. The DMF with mild reductive ability plays a key role in the formation of such a novel cluster-in-cluster silver nanocluster. The fcc-structured Ag_10_ kernel is built from two single-edge opened Ag_6_ octahedra by sharing one edge and further locked by a pair of Mo_7_O_26_^10–^ anions to form an inner Ag_10_@(Mo_7_O_26_)_2_ core which is finally encapsulated by an outer Ag_70_ shell to form three-shell Ag_10_@(Mo_7_O_26_)_2_@Ag_70_ nanoclusters. Notably, both the bioctahedral Ag_10_ kernel and crescent-like Mo_7_O_26_^10–^ have not been observed in silver nanocluster and POM chemistry ever before, respectively. The bioctahedral Ag_10_ core can be deemed as a brand-new embryo state of silver nanoparticles; moreover, it also provides a new edge-fusion growth route for silver nanoparticles from the smallest Ag_6_ nanofragment of metallic silver. We hope that this work can popularize the new controllable synthetic method to expand the scope of silver nanoclusters with higher complexity.

## Conflicts of interest

There are no conflicts to declare.

## Supplementary Material

Supplementary information

Crystal structure data
