# Supplementary material for: Unusual fcc-structured Ag10 kernels trapped in Ag70 nanoclusters
Source: Chem Sci. 2018 Oct 18;10(2):564–8. doi: 10.1039/c8sc03396j (PMC6333236; doi:10.1039/c8sc03396j)
Supplement: Supplementary file 1 [file SC-010-C8SC03396J-s001.pdf]

Electronic Supplementary Material (ESI) for Chemical Science.
This journal is © The Royal Society of Chemistry 2018

Supporting Information (SI)

Unusual *fcc*-Structured Ag_{10} Kernel Trapped in Ag_{70} Nanocluster

Yan-Min Su,[⊥,a] Zhi Wang,[⊥,a] Gui-Lin Zhuang,[b] Quan-Qin Zhao,[a] Xing-Po Wang,[a] Chen-Ho Tung,[a] and Di Sun[*,a]

[a]Key Lab of Colloid and Interface Chemistry, Ministry of Education, School of Chemistry and Chemical Engineering, Shandong University, Jinan, 250100, P. R. China. Email: dsun@sdu.edu.cn

[b]College of Chemical Engineering and Materials Science, Zhejiang University of Technology, Hangzhou, 310032, People's Republic of China.

Experimental details

The precursor of $(Ag^{i}PrS)_n$ was prepared by the following reported procedure[1]. The synthesis of $(CyhSAg)_n$ was similar to that of $(Ag^{i}PrS)_n$, except that the ^{i}PrSH was replaced by CyhSH. All reagents employed were commercially available and used as received without further purification. The solvents were purified and distilled by standard procedures prior to use. IR spectrum was recorded on a Bruker ALPHA in the frequency range of 4000-400 cm^{-1}. The elemental analyses (C, H and N) were determined on a Vario EL III analyzer. Morphology of the samples and elemental composition analyses were measured using an SU-8010 field emission scanning electron microscope (FESEM; Hitachi Ltd., Tokyo, Japan) equipped with an Oxford-Horiba Inca XMax50 energy dispersive X-ray spectroscopy (EDS) attachment (Oxford Instruments Analytical, High Wycombe, England). Powder X-ray diffraction (PXRD) data were collected on a Philips X'Pert Pro MPD X-ray diffractometer with CuKα radiation equipped with an X'Celerator detector. The diffuse-reflectance spectra were recorded on a UV/Vis spectrophotometer (Evolution 220, ISA-220 accessory, Thermo Scientific) using a built-in 10 mm silicon photodiode with a 60 mm Spectralon sphere. Temperature-dependent photoluminescence measurements were carried out in an Edinburgh spectrofluorimeter (F920S) coupled with an Optistat DN cryostat (Oxford Instruments), and the ITC temperature controller and a pressure gauge were used to realize the variable-temperature measurement in the range of 83-293 K. Spectra were collected at different temperatures after a 2 min homoiothermy. Time-resolved photoluminescence lifetime measurement was measured on Edinburgh spectrofluorimeter (F920S) using a time-correlated single-photon counting technique. Electrochemical measurements were performed with a CHI660E electrochemical workstation. A conventional three-electrode system was used. The working electrode was a carbon paste electrode (CPE), a Pt wire was used as the counter electrode, and an Ag/AgCl (3 M KCl) electrode was used as the reference electrode. The CPE was prepared as follows: graphite powder (0.1 g) and an aliquot of the sample (0.01 g) were mixed and ground together by a gate mortar and pestle to achieve a dry mixture; to the mixture paraffin oil (0.05 mL) was added under stirring with a glass rod. Then the mixture was packed into a 3 mm inner diameter polytetrafluoroethylene tube, and the surface was pressed tightly onto weighing

paper with a copper rod through the back. Electrical contact was established with a copper rod through the back of the electrode.

X-ray Crystallography

Single crystals of **SD/Ag80a** and **SD/Ag80b** with appropriate dimensions were chosen under an optical microscope and quickly coated with high vacuum grease (Dow Corning Corporation) to prevent decomposition. Intensity data and cell parameters of **SD/Ag80a** (83 K) and **SD/Ag80b** (100 K) were recorded on a Rigaku XtaLAB Synergy diffractometer coupled to a Rigaku Hypix detector with Mo Kα radiation (λ = 0.71073 Å) from PhotonJet micro-focus X-ray sources. The diffraction images for **SD/Ag80a** and **SD/Ag80b** were processed and scaled using the CrysAlisPro software[2] The structures were solved using the charge-flipping algorithm, as implemented in the program SUPERFLIP[3] and refined by full-matrix least-squares techniques against F_o^2 using the SHELXL program[4] through the OLEX2 interface.[5] Hydrogen atoms at carbon were placed in calculated positions and refined isotropically by using a riding model. Appropriate restraints or constraints were applied to the geometry and the atomic displacement parameters of the atoms in the cluster. All structures were examined using the Addsym subroutine of PLATON[6] to ensure that no additional symmetry could be applied to the models. Pertinent crystallographic data collection and refinement parameters are collated in Table S3. Selected bond lengths and angles are collated in Table S4.

Synthesis

Synthesis of SD/Ag80a.

$(CyhSAg)_n$ (5.6 mg, 0.025 mmol) and $Na_2MoO_4{\cdot}2H_2O$ (5.0 mg, 0.0207 mmol) were mixed in 6 mL CH_3OH-DCM-^{n}PrOH (v:v:v = 1:1:1), and CF_3SO_3Ag (12.8 mg, 0.05 mmol, dissolved in 100 μL DMF) was added to the above solution. The resulting suspension was sealed in a 25 mL Teflon-lined reaction vessel and heated at 65 °C for 2000 min. After cooling, the pale brown solution was filtered and evaporated slowly in the dark at room temperature. **SD/Ag80a** crystallized as brown block crystals after 1-2 weeks. Anal. Calc. (found) for **SD/Ag80a**: ($C_{268}H_{484}Ag_{80}F_{48}Mo_{16}N_8O_{120}S_{52}$): C, 17.42 (17.45); H, 2.64 (2.61); N, 0.60 (0.63) %. Selected IR peaks (cm^{-1}) of **SD/Ag80a**: 3000 (m), 1450 (w), 1255 (w), 1220 (s), 1150 (m), 1020 (s), 780 (s), 710 (w), 630 (s), 560 (w), 505 (m).

Synthesis of SD/Ag80b.

$(^iPrSAg)_n$ (9.2 mg, 0.05 mmol) and $(^nBu_4N)_2(Mo_6O_{19})$ (5 mg, 0.0037 mmol) were mixed in 4.5 mL CH_3OH-DMF (v:v = 8:1), and CF_3SO_3Ag (25.7 mg, 0.1 mmol, dissolved in 100 μL DMF) was added to the above solution. The resulting suspension was sealed in a 25 mL Teflon-lined reaction vessel and heated at 65 °C for 2000 min. After cooling, the brown solution was filtered and evaporated slowly in the dark at room temperature. Red block crystals of **SD/Ag80b** were crystallized after 1-2 weeks. Anal. Calc. (found) for **SD/Ag80b**: ($C_{142}H_{294}Ag_{80}F_{48}Mo_{16}N_6O_{114}S_{52}$): C, 10.24 (10.28); H, 1.78 (1.75); N, 0.50 (0.52) %.

Figure S1: The structural diagrams of SD/Ag80b.

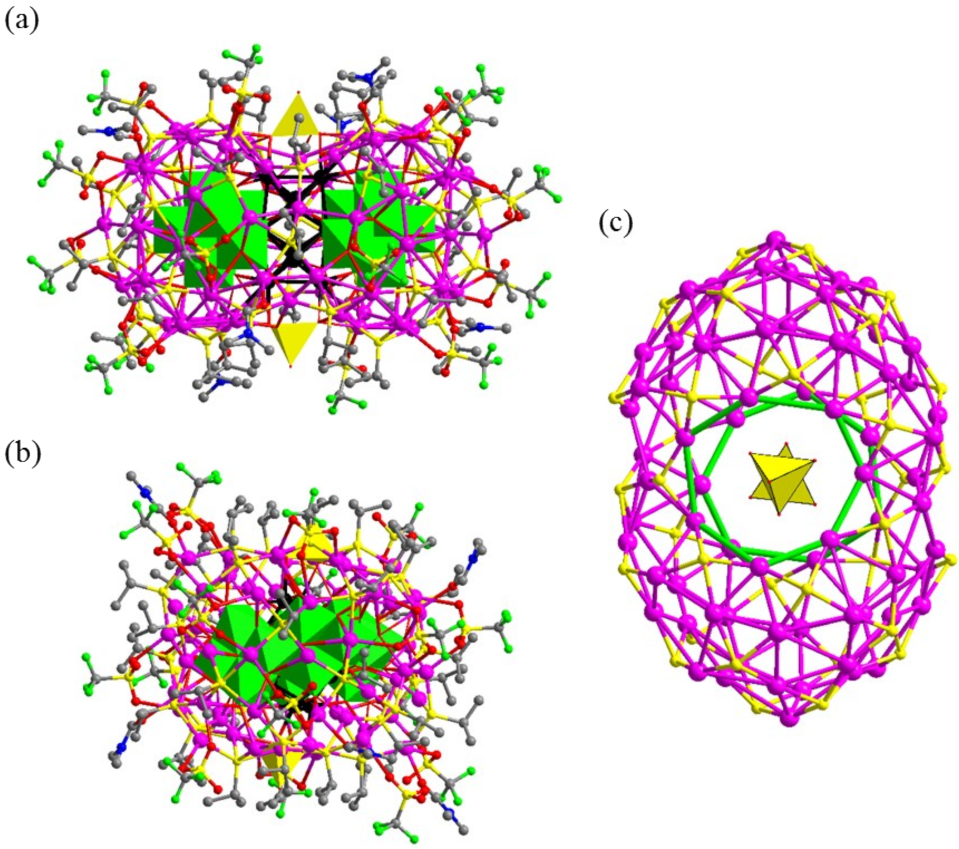

(a) and (b) The X-ray crystal structure of $Ag_{10}@(Mo_7O_{26})_2@Ag_{70}$ nanocluster viewed along two orthogonal directions. The inner silver atoms of Ag_{10} kernel are highlighted by black. The $Mo_7O_{26}^{10-}$ and MoO_4^{2-} are represented by green and yellow polyhedral, respectively. (c) The $Ag_{70}S_{36}$ shell with silver heptagons highlighted by green.

Figure S2: The Ag···Ag distances in Ag_6 octahedron of SD/Ag80a.

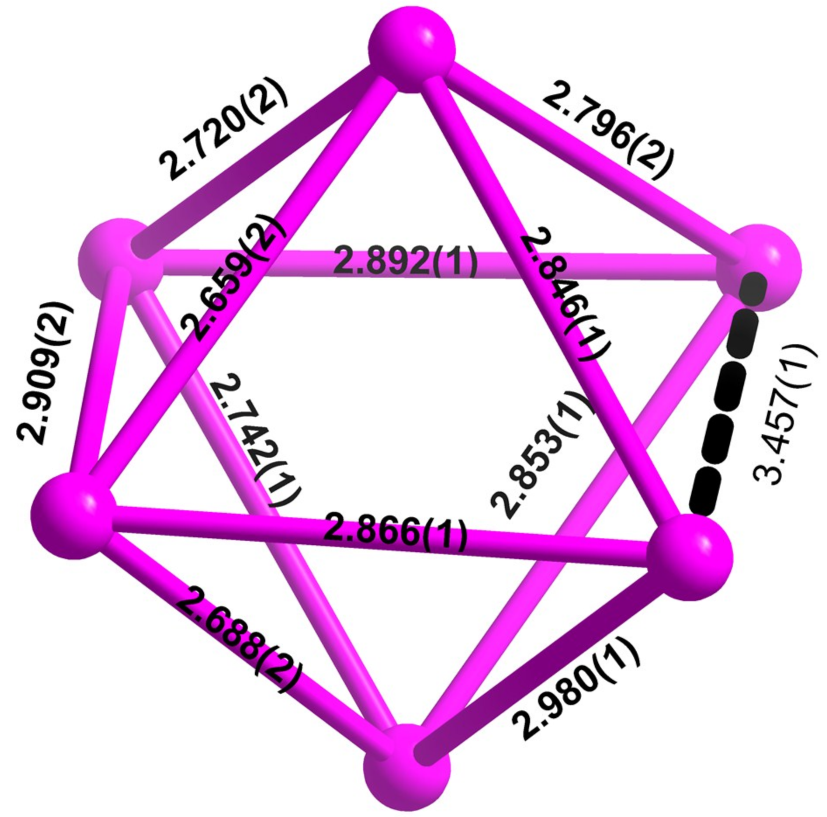

Figure S3: The argentophilic interactions between Ag_{10} kernel and Ag_{70} shell.

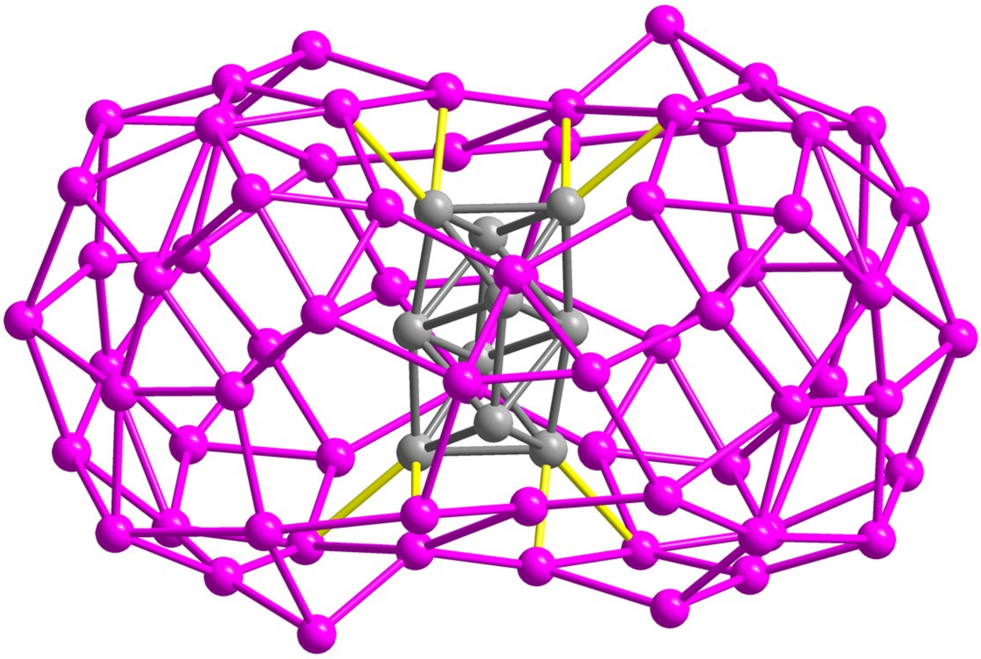

Figure S4: The ^{13}C NMR of HCl digested reaction mother solution of SD/Ag80a.

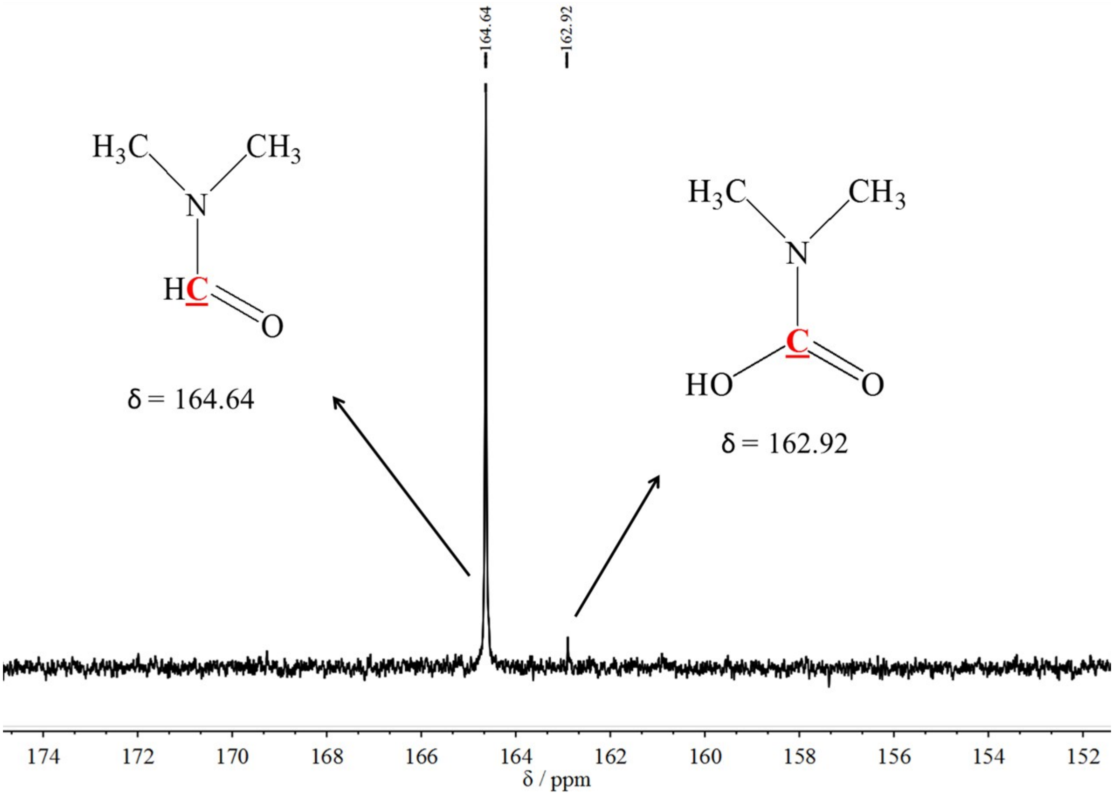

^{13}C NMR spectrum was recorded in a J. Young NMR tube on Bruker Avance 500 spectrometers. (In general, 100 μL concentrated mother solution of **SD/Ag80a** was digested with 10 μL HCl (37%), then 400 μL CD_3OD was added to the above solution. The digestion solution was used directly for ^{13}C NMR measurement.) The chemical shifts are reported in parts per million δ (ppm) referenced to the residual proton signal of the deuterated solvent.

Figure S5: A_g**-symmetry HOMO-2 (a),** A_u**-symmetry HOMO-1 (b),** A_u**-symmetry HOMO (c),** A_u**--symmetry LUMO (d)**

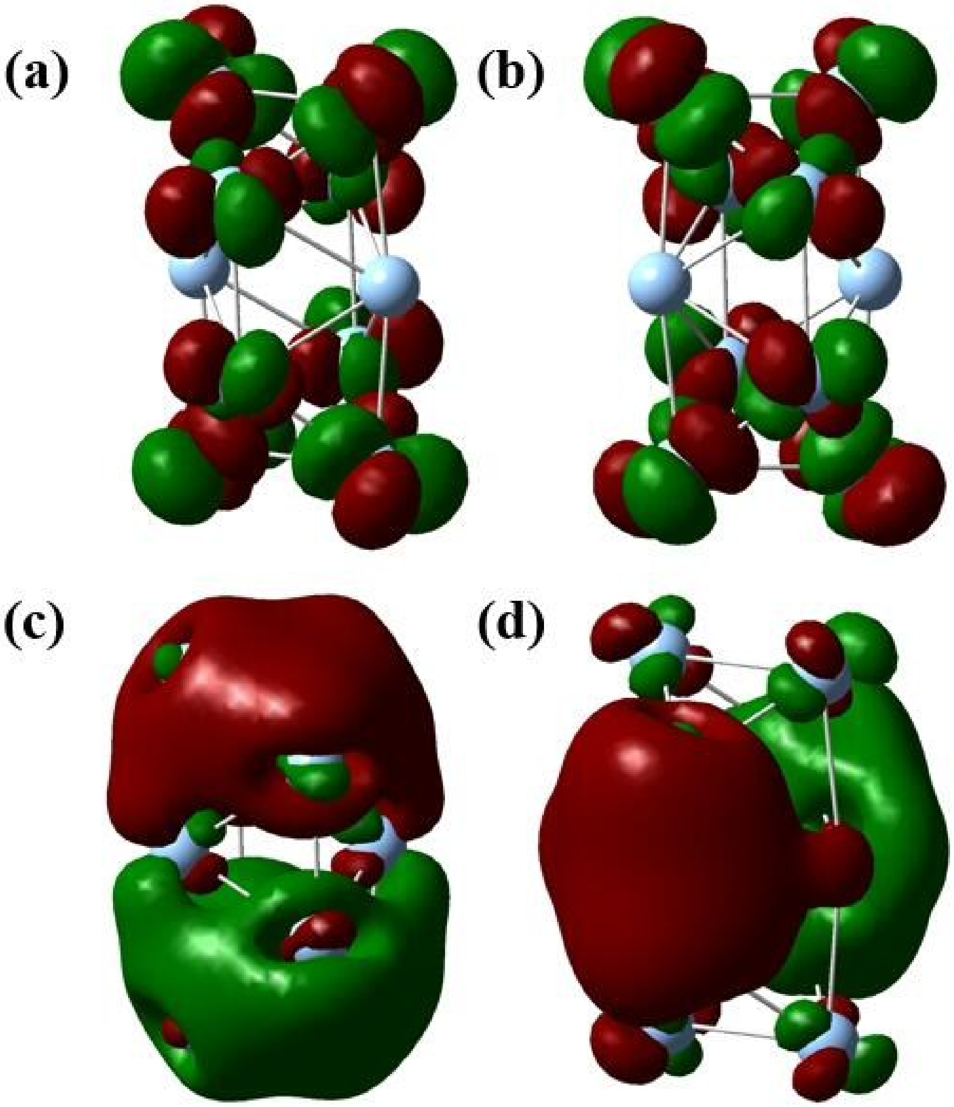

Computational details:

The initial structure is derived from single-crystal diffraction result. Due to the huge computational cost of Ag_{80}, it is very difficult to conduct ab-initio calculation toward the whole cluster. Fortunately, the electronic structure of inner Ag_{10} kernel is very isolated because of the geometrical separation of O-bridges from other Ag_{70} outer layer. Thus, inner Ag_{10} kernel was taken into account in the DFT calculations. Density functional theory calculation were performed in theoretical level of B3LYP/SDD within the software Gaussian 03 program.

Figure S6: UV-Vis spectra of Kubelka-Munk function vs energy (eV) of SD/Ag80a and (CyhSAg)$_n$.

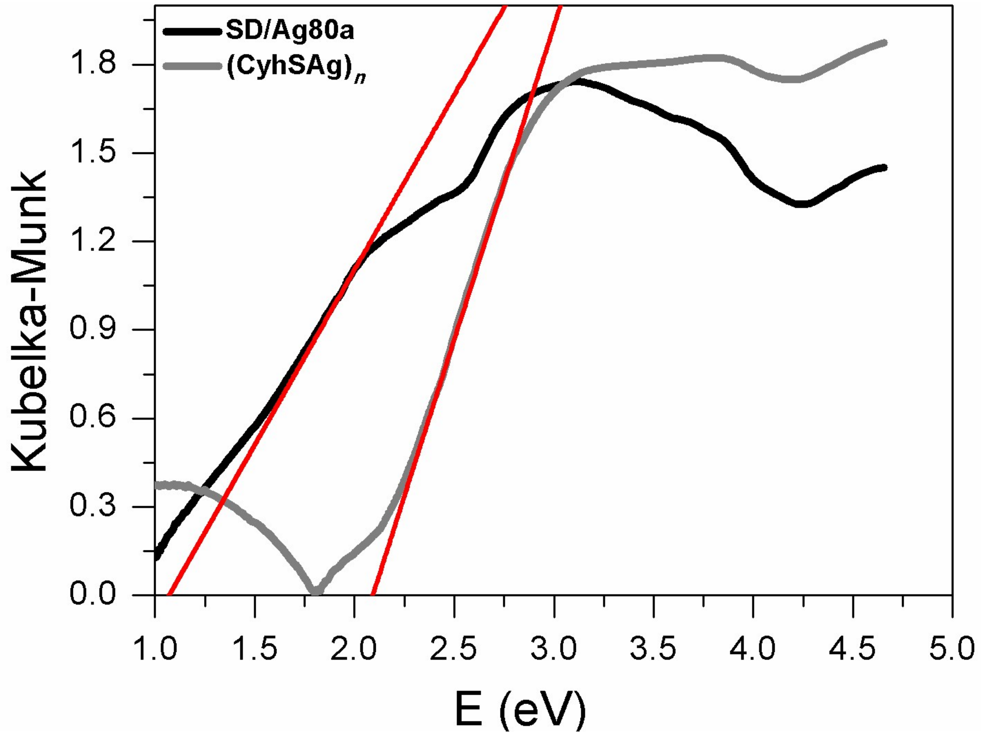

Figure S7: The luminescence decay curve of SD/Ag80a at 83 K.

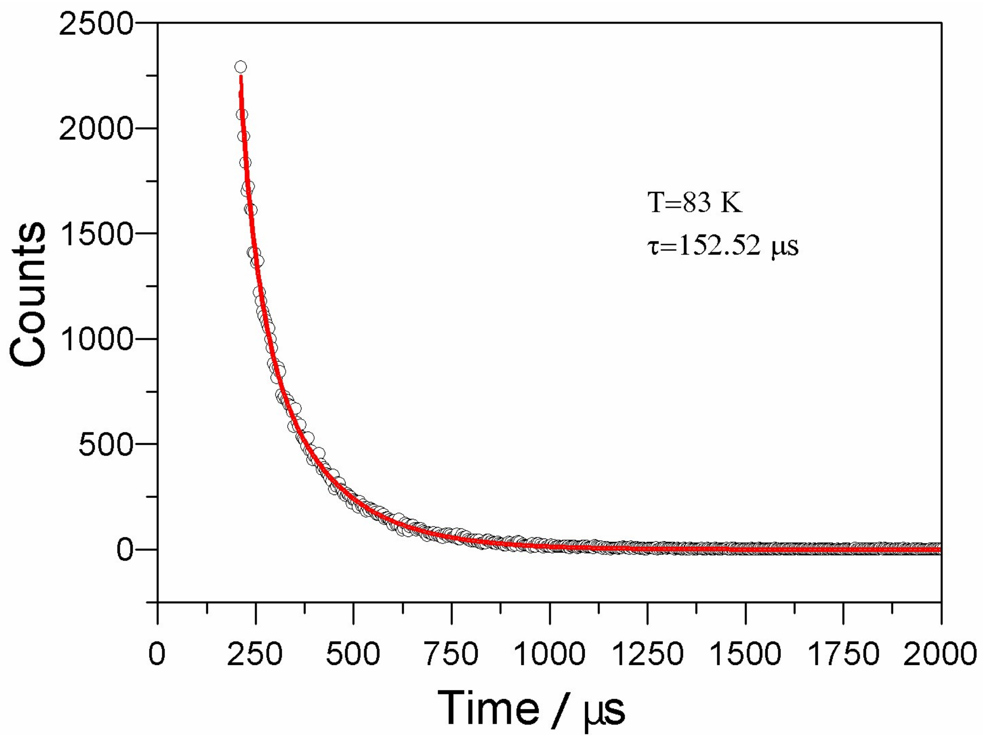

Figure S8: Powder X-ray diffraction (PXRD) patterns of SD/Ag80a (top) and SD/Ag80b (down).

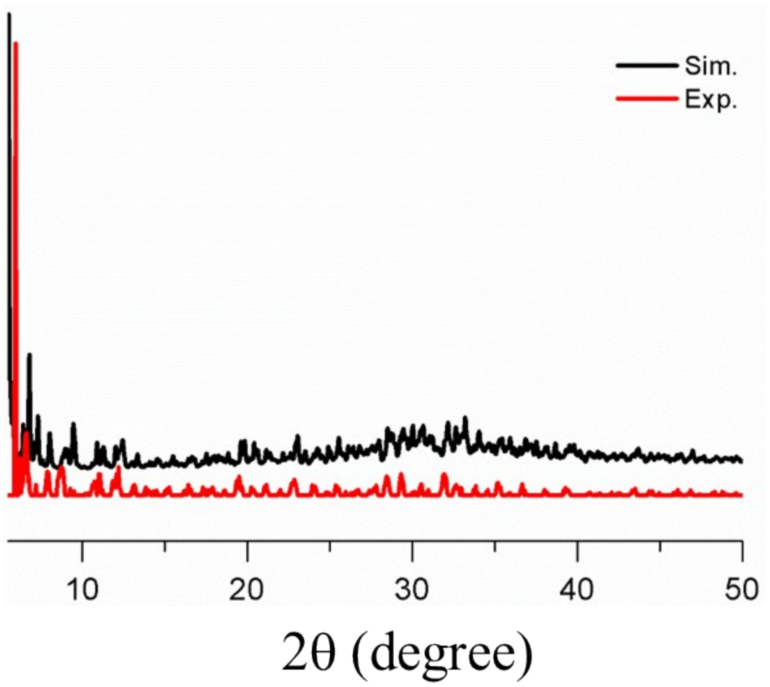

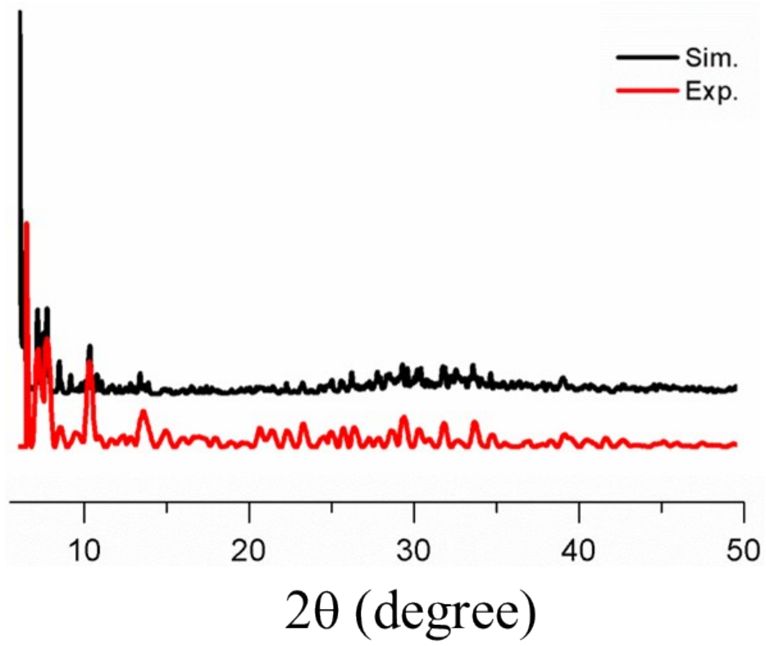

Figure S9: IR spectrum of SD/Ag80a.

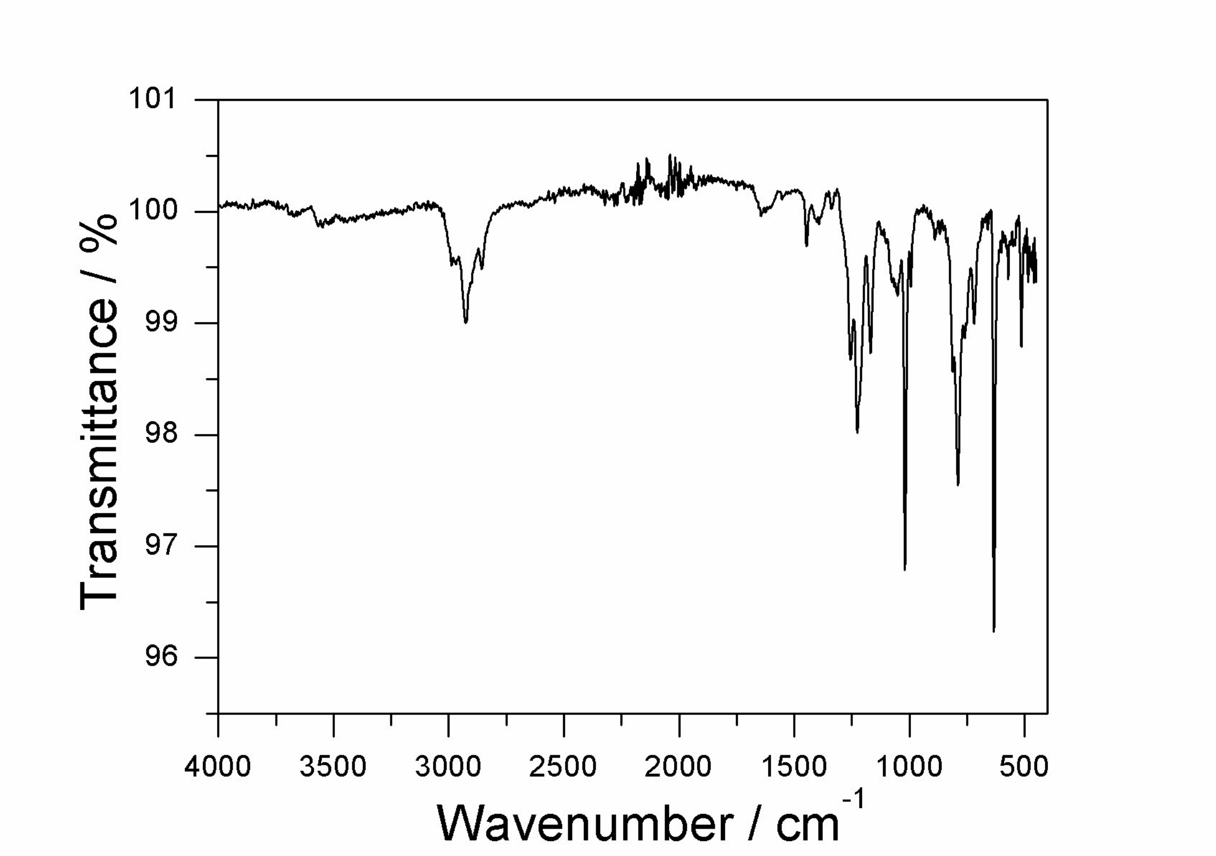

Figure S10: The cycle voltammogram of SD/Ag80a measured in 0.5 M H_2SO_4 solution at 0.1 V s^{-1} using its carbon pasted electrode.

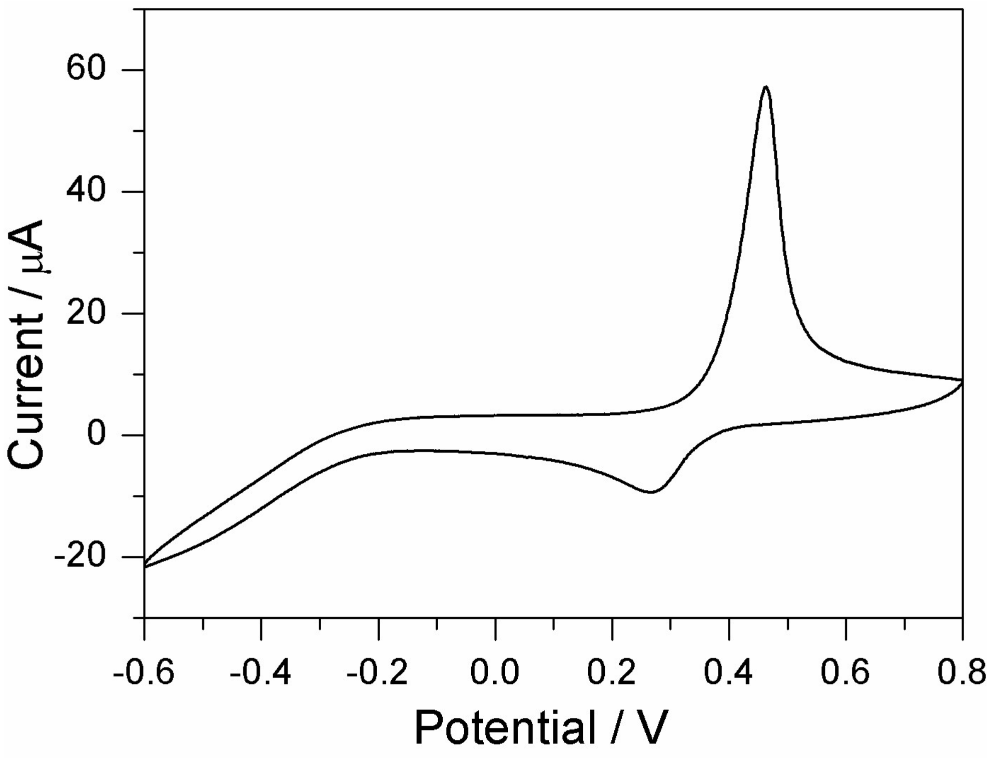

Figure S11: SEM and elemental mapping images of SD/Ag80a.

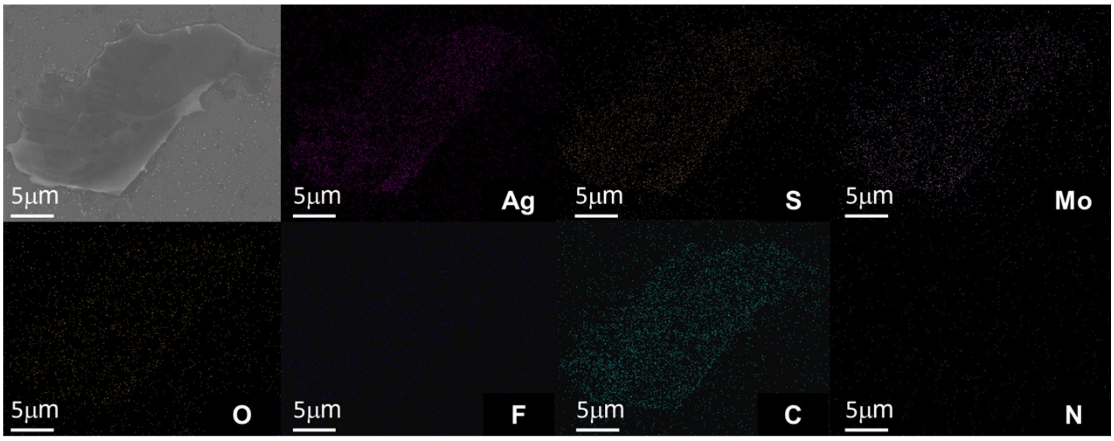

Table S1: Summary of reaction conditions tried in the synthesis of SD/Ag80a.

Precursor	Silver salt	Template	Solvent	Phenomenon
$(CyhSAg)_n$	$AgNO_3$	$Na_2MoO_4 \cdot 2H_2O$	MeOH:DCM:^{n}PrOH:DMF	colorless solution
	AgOAc			yellow solution
	$AgCOOCF_3$			pale yellow solution
	$AgSO_3CH_3$			pale yellow solution
	PhCOOAg			pale yellow solution
	p-TOSAg			yellow solution
	$AgSO_3CF_3$	$Na_2MoO_4 \cdot 2H_2O$	MeOH:DCM:^{n}PrOH:DMF	**SD/Ag80a**
		$(^nBu_4N)_2(Mo_6O_{19})$		brown solution
		$(NH_4)_6Mo_7O_{24}$		pale brown solution
		$(^nBu_4N)_4[\alpha\text{-}Mo_8O_{26}]$		orange solution
	$AgSO_3CF_3$	$Na_2MoO_4 \cdot 2H_2O$	MeOH:DCM:DMF	pale brown solution
			MeOH:DMF	pale brown solution
			MeOH:DCM:^{i}PrOH	pale brown solution
			MeCN:DCM:^{n}PrOH:DMF	pale yellow solution
			MeOH:DMF:^{n}PrOH	colorless solution

Table S2: Summary of reaction conditions tried in the synthesis of SD/Ag80b.

Precursor	Silver salt	Template	Solvent	Phenomenon
$(^{i}PrSAg)_n$	$AgSO_3CF_3$	$(^{n}Bu_4N)_2(Mo_6O_{19})$	MeOH:DMF	**SD/Ag80b**
			MeCN:DMF	orange solution
			MeOH:DCM:^{n}PrOH:DMF	brown solution
			MeOH:DMF:^{n}PrOH	colorless solution
		$Na_2MoO_4 \cdot 2H_2O$	MeCN:DMF	yellow solution
			MeOH:DCM:^{n}PrOH:DMF	colorless solution
			MeOH:DMF:^{n}PrOH	colorless solution
		$(^{n}Bu_4N)_4[\alpha\text{-}Mo_8O_{26}]$	MeOH:DMF	**SD/Ag9**[10]
			MeOH:DCM:^{n}PrOH:DMF	brown solution
			MeOH	brown solution
			MeCN	yellow solution
			MeOH:DCM:DMF	brown solution
			MeCN:DMAc	brown solution
			MeOH:DMF:^{n}PrOH	colorless solution
		$(NH_4)_6Mo_7O_{24}$	MeOH:DMF	brown solution
			MeCN:DMF	yellow solution
			MeOH:DCM:^{n}PrOH:DMF	**SD/Ag12**[10]
	CF_3COOAg	$Na_2MoO_4 \cdot 2H_2O$	MeCN	colorless solution
			MeOH:DMF	yellow solution
			MeOH:DCM:DMF	colorless solution
			MeCN:DMF	colorless solution
		$(^{n}Bu_4N)_2(Mo_6O_{19})$	MeCN	yellow solution
			MeOH:DMF	orange solution
			MeOH:DCM:DMF	brown solution
			MeCN:DMF	brown solution
		$(NH_4)_6Mo_7O_{24}$	MeCN	colorless solution
			MeOH:DMF	colorless solution
			MeCN:DMF	yellow solution
		$(^{n}Bu_4N)_4[\alpha\text{-}Mo_8O_{26}]$	MeCN	yellow solution
			MeOH:DMF	orange solution

			MeOH:DCM:DMF	yellow solution
			MeCN:DMF	yellow solution
	$AgBF_4$	$Na_2MoO_4 \cdot 2H_2O$	MeOH:DMF	brown solution
			MeCN	colorless solution
			MeOH:DCM:DMF	yellow solution
			MeCN:DMF	yellow solution
		$(^nBu_4N)_2(Mo_6O_{19})$	MeOH:DMF	brown solution
			MeCN	yellow solution
			MeCN:DMF	orange solution
		$(NH_4)_6Mo_7O_{24}$	MeOH:DMF	brown solution
			MeCN	pale blue solution
			MeOH:DCM:DMF	brown solution
			MeCN:DMF	yellow solution
		$(^nBu_4N)_4[\alpha\text{-}Mo_8O_{26}]$	MeOH:DMF	brown solution
			MeCN	yellow solution
			MeOH:DCM:DMF	brown solution
			MeCN:DMF	yellow solution
	p-TOSAg	$Na_2MoO_4 \cdot 2H_2O$	MeOH:DMF	red solution
			MeCN	colorless solution
			MeOH:DCM:DMF	brown solution
			MeCN:DMF	colorless solution
			MeOH	brown solution
		$(^nBu_4N)_2(Mo_6O_{19})$	MeOH:DMF	orange solution
			MeCN	yellow solution
			MeOH:DCM:DMF	brown solution
			MeCN:DMF	colorless solution
			MeOH	yellow solution
		$(NH_4)_6Mo_7O_{24}$	MeOH:DMF	orange solution
			MeCN	yellow solution
			MeOH:DCM:DMF	brown solution
			MeCN:DMF	yellow solution
			MeOH	yellow solution
		$(^nBu_4N)_4[\alpha\text{-}Mo_8O_{26}]$	MeOH:DMF	**SD/Ag7**[10] and **SD/Ag8**[10]
			MeCN	**SD/Ag12**[10]
			MeOH:DCM:	brown solution

			DMF	
			MeCN:DMF	colorless solution
			MeOH	**SD/Ag11**[10]
	$AgNO_3$	$Na_2MoO_4 \cdot 2H_2O$	MeOH:DMF	colorless solution
		$(^nBu_4N)_2(Mo_6O_{19})$		colorless solution
		$(NH_4)_6Mo_7O_{24}$		colorless solution
		$(^nBu_4N)_4[\alpha\text{-}Mo_8O_{26}]$		**SD/Ag10**[10]
	AgOAc	$Na_2MoO_4 \cdot 2H_2O$	MeOH:DMF	colorless solution
			MeCN	colorless solution
			MeOH:DCM: DMF	yellow solution
		$(^nBu_4N)_2(Mo_6O_{19})$	MeCN:DMF	brown solution
			MeCN	colorless solution
			MeOH:DCM: DMF	colorless solution
		$(NH_4)_6Mo_7O_{24}$	MeCN:DMF	colorless solution
			MeCN	colorless solution
			MeOH:DCM: DMF	colorless solution
		$(^nBu_4N)_4[\alpha\text{-}Mo_8O_{26}]$	MeCN:DMF	colorless solution
			MeCN	colorless solution
			MeOH:DCM: DMF	colorless solution

Table S3: Crystal data and structure refinements for SD/Ag80a and SD/Ag80b.

Identification code	**SD/Ag80a**	**SD/Ag80b**
Empirical formula	$C_{268}H_{484}Ag_{80}F_{48}Mo_{16}N_8O_{120}S_{52}$	$C_{142}H_{294}Ag_{80}F_{48}Mo_{16}N_6O_{114}S_{52}$
Formula weight	18482.36	16653.57
Temperature/K	83.0(6)	100.00(10)
Crystal system	monoclinic	triclinic
Space group	$P2_1$/n	P-1
a/Å	25.1615(4)	21.9533(3)
b/Å	30.4928(5)	22.1690(3)
c/Å	32.0095(9)	22.9990(3)
α/°	90	111.8795(11)
β/°	90.675(2)	109.2284(11)
γ/°	90	92.0299(10)
Volume/Å^3	24557.4(9)	9646.4(2)
Z	2	1
ρ_{calc}g/cm^3	2.500	2.867
μ/mm^{-1}	3.798	4.816
F(000)	17608.0	7796.0
Radiation	MoKα (λ = 0.71073)	MoKα (λ = 0.71073)
Reflections collected	176090	100166
Independent reflections	43585 [R_{int} = 0.0674, R_{sigma} = 0.0544]	34057 [R_{int}= 0.0391, R_{sigma}= 0.0442]
Data/restraints/parameters	43585/289/2666	34057/486/2248
Goodness-of-fit on F^2	1.069	2.247
Final R indexes [I>=2σ (I)]	R_1= 0.0737, wR_2 = 0.1725	R_1= 0.0444, wR_2= 0.0923
Final R indexes [all data]	R_1= 0.1081, wR_2 = 0.2016	R_1= 0.0584, wR_2= 0.0943
Largest diff. peak/hole / e Å^{-3}	4.75/-3.24	3.10/-2.23

Table S4: Selected bond distances (Å) and angles (°) for SD/Ag80a and SD/Ag80b.

SD/Ag80a			
Ag1—Ag14^{i}	2.862(2)	Ag24—S7	2.427(4)
Ag1—S10	2.489(5)	Ag24—S12	2.420(4)
Ag1—S11	2.603(4)	Ag25—Ag9^{i}	3.1818(17)
Ag1—O12	2.490(14)	Ag25—Ag13^{i}	3.0835(19)
Ag1—O46	2.477(11)	Ag25—Ag26	3.1099(19)
Ag2—Ag3	3.142(2)	Ag25—S12	2.618(4)
Ag2—Ag40	3.2065(18)	Ag25—S13	2.476(4)
Ag2—S8	2.461(4)	Ag25—O27^{i}	2.505(11)
Ag2—S10	2.407(5)	Ag25—O47	2.363(9)
Ag2—O37	2.541(9)	Ag26—Ag13^{i}	2.833(2)
Ag3—Ag4	3.058(2)	Ag26—S11	2.452(5)
Ag3—Ag31	3.255(2)	Ag26—S12	2.431(5)
Ag3—S10	2.442(4)	Ag26—O46	2.435(9)
Ag3—S14	2.536(5)	Ag27—Ag35	3.254(2)
Ag3—O34	2.458(14)	Ag27—S2	2.479(4)
Ag4—Ag5	3.076(2)	Ag27—S20	2.469(5)
Ag4—Ag6	3.2410(18)	Ag27—O1	2.43(3)
Ag4—Ag39^{i}	3.3038(18)	Ag27—O18	2.542(19)
Ag4—S14	2.570(4)	Ag28—Ag31	3.2781(19)
Ag4—S22	2.395(4)	Ag28—Ag36	3.2145(19)
Ag4—O35	2.341(11)	Ag28—S8	2.454(4)
Ag5—Ag37^{i}	2.8523(19)	Ag28—S16	2.517(4)
Ag5—S22	2.399(5)	Ag28—O7	2.492(12)
Ag5—S25^{i}	2.722(5)	Ag29—Ag30	3.1912(18)
Ag5—O25	2.251(11)	Ag29—Ag34	3.3224(18)
Ag6—Ag7	2.7201(16)	Ag29—S16	2.422(4)
Ag6—Ag8	3.2969(15)	Ag29—S20	2.452(5)
Ag6—Ag37^{i}	2.9090(16)	Ag29—O40	2.510(10)
Ag6—Ag38	2.8920(15)	Ag30—Ag31	2.9897(17)
Ag6—Ag39^{i}	2.7424(14)	Ag30—S16	2.475(4)
Ag6—O28	2.490(11)	Ag30—S17	2.476(4)
Ag6—O35	2.433(10)	Ag30—O60	2.486(9)
Ag6—O53	2.358(10)	Ag31—S14	2.445(4)
Ag7—Ag37^{i}	2.6588(17)	Ag31—S16	2.459(4)
Ag7—Ag38	2.7954(15)	Ag31—O36	2.511(10)
Ag7—Ag38^{i}	2.8465(14)	Ag32—Ag34	3.0756(17)
Ag7—Ag39	2.8882(15)	Ag32—S17	2.455(4)
Ag7—O47^{i}	2.284(10)	Ag32—S19	2.467(4)
Ag7—O52	2.309(10)	Ag32—O5	2.583(10)

Ag8—Ag11	3.3289(17)	Ag33—Ag34	3.1291(17)
Ag8—Ag30	2.9815(17)	Ag33—Ag35	3.0148(19)
Ag8—Ag31	2.9681(17)	Ag33—S4	2.578(4)
Ag8—S14	2.476(4)	Ag33—S19	2.530(4)
Ag8—S17	2.557(4)	Ag33—O58	2.558(11)
Ag8—O28	2.454(11)	Ag33—O59	2.440(9)
Ag8—O53	2.437(10)	Ag34—Ag35	2.9574(18)
Ag9—Ag22^{i}	3.1262(19)	Ag34—S19	2.509(4)
Ag9—Ag24^{i}	3.143(2)	Ag34—S20	2.507(5)
Ag9—Ag25^{i}	3.1819(17)	Ag34—O60	2.447(10)
Ag9—S12^{i}	2.511(4)	Ag35—S4	2.469(5)
Ag9—S23	2.487(4)	Ag35—S20	2.467(4)
Ag9—O27	2.424(11)	Ag36—Ag40	3.0786(18)
Ag10—Ag18^{i}	3.0107(19)	Ag36—S2	2.507(5)
Ag10—Ag20^{i}	2.9823(18)	Ag36—S8	2.453(4)
Ag10—Ag37^{i}	3.3621(18)	Ag37—Ag5^{i}	2.8523(19)
Ag10—S23	2.476(4)	Ag37—Ag6^{i}	2.9089(16)
Ag10—S25^{i}	2.488(5)	Ag37—Ag7^{i}	2.6587(17)
Ag10—O25	2.454(11)	Ag37—Ag10^{i}	3.3621(18)
Ag10—O42^{i}	2.515(10)	Ag37—Ag38	2.8665(15)
Ag11—Ag12	3.2245(18)	Ag37—Ag39	2.6879(16)
Ag11—Ag32	3.2767(17)	Ag37—O25^{i}	2.422(11)
Ag11—S13^{i}	2.486(4)	Ag37—O42	2.306(10)
Ag11—S17	2.584(4)	Ag37—O48	2.380(10)
Ag11—O28	2.568(11)	Ag38—Ag7^{i}	2.8465 (14)
Ag12—Ag13	3.1891(17)	Ag38—Ag39^{i}	2.8525(15)
Ag12—Ag32	2.9118(17)	Ag38—Ag39	2.9805(15)
Ag12—S13^{i}	2.444(4)	Ag38—O38	2.351(9)
Ag13—Ag25^{i}	3.0835(19)	Ag38—O51	2.341(9)
Ag13—Ag26^{i}	2.833(2)	Ag39—Ag4^{i}	3.3036(18)
Ag13—S11^{i}	2.621(4)	Ag39—Ag6^{i}	2.7425(14)
Ag13—S13^{i}	2.550(4)	Ag39—Ag38^{i}	2.8526(15)
Ag13—O33	2.413(16)	Ag39—O45^{i}	2.334(10)
Ag13—O57	2.466(10)	Ag39—O50	2.311(10)
Ag14—Ag1^{i}	2.862(2)	Ag40—S7	2.608(4)
Ag14—Ag15	3.100(2)	Ag40—S8	2.648(4)
Ag14—S11^{i}	2.450(4)	Ag40—O37	2.495(10)
Ag14—S22^{i}	2.445(4)	Mo1—O37	2.162(9)
Ag14—O50	2.425(10)	Mo1—O38	2.074(9)
Ag15—Ag17	3.006(2)	Mo1—O44	2.204(10)
Ag15—S21	2.389(5)	Mo1—O45	1.778(10)
Ag15—O23	2.406(15)	Mo1—O46	1.770(9)
Ag15—O57	2.210(10)	Mo1—O47	1.776(9)

Ag16—Ag17	2.8998(19)	Mo2—O35	1.748(10)
Ag16—Ag18	3.286(2)	Mo2—O36	1.743(10)
Ag16—S21	2.415(5)	Mo2—O37	1.854(9)
Ag16—S25	2.413(5)	Mo2—O38	2.227(9)
Ag17—Ag33	3.0020(18)	Mo2—O39	1.956(9)
Ag17—S4	2.428(4)	Mo2—O41	2.293(9)
Ag17—S21	2.427(5)	Mo3—O25	1.810(11)
Ag18—Ag10^{i}	3.0107(19)	Mo3—O26	1.723(11)
Ag18—Ag19	3.1462(19)	Mo3—O27	1.742(12)
Ag18—Ag20	2.9306(19)	Mo3—O28	1.773(12)
Ag18—S24	2.468(4)	Mo4—O39	1.956(9)
Ag18—S25	2.434(4)	Mo4—O51	2.241(8)
Ag18—O55	2.399(10)	Mo4—O53	1.759(10)
Ag19—Ag35	3.259(2)	Mo4—O56	2.192(10)
Ag19—S4	2.505(4)	Mo4—O58	1.825(10)
Ag19—S24	2.513(4)	Mo4—O60	1.759(9)
Ag19—O17	2.465(14)	Mo5—O48	1.746(9)
Ag19—O19	2.600(16)	Mo5—O49	1.956(9)
Ag20—Ag10^{i}	2.9824(18)	Mo5—O51	2.183(10)
Ag20—Ag21	3.3300(18)	Mo5—O55	1.741(11)
Ag20—Ag22	3.2886(19)	Mo5—O56	2.353(9)
Ag20—S23^{i}	2.508(4)	Mo5—O59	1.861(9)
Ag20—S24	2.471(4)	Mo6—O39	2.172(9)
Ag20—O43	2.422(10)	Mo6—O40	1.739(10)
Ag21—Ag23	2.9961(19)	Mo6—O41	1.935(9)
Ag21—S2	2.458(5)	Mo6—O49	2.197(9)
Ag21—S24	2.473(5)	Mo6—O54	1.728(10)
Ag21—O15	2.519(14)	Mo6—O56	1.937(9)
Ag21—O16	2.526(17)	Mo7—O38	2.163(9)
Ag22—Ag9^{i}	3.1262(19)	Mo7—O41	2.219(9)
Ag22—Ag24	3.110(2)	Mo7—O42	1.757(10)
Ag22—S7	2.448(5)	Mo7—O43	1.764(9)
Ag22—S23^{i}	2.501(5)	Mo7—O44	1.832(10)
Ag22—O31	2.390(13)	Mo7—O49	1.988(9)
Ag23—Ag36	2.9241(18)	Mo8—O50	1.765(9)
Ag23—Ag40	3.1158(19)	Mo8—O51	2.046(9)
Ag23—S2	2.455(4)	Mo8—O52	1.777(11)
Ag23—S7	2.467(4)	Mo8—O57	1.797(10)
Ag23—O43	2.373(10)	Mo8—O58	2.208(9)
Ag24—Ag9^{i}	3.143(2)	Mo8—O59	2.165(10)
Ag24—Ag40	3.1554(17)		
S10—Ag1—S11	154.72(16)	S8—Ag36—S2	151.71(14)
S10—Ag1—O12	91.8(3)	O42—Ag37—O25^{i}	85.3(3)

O12—Ag1—S11	102.6(3)	O42—Ag37—O48	86.0(3)
O46—Ag1—S10	117.7(3)	O48—Ag37—O25^{i}	98.7(4)
O46—Ag1—S11	85.0(2)	O51—Ag38—O38	100.2(3)
O46—Ag1—O12	83.0(4)	O50—Ag39—O45^{i}	76.4(3)
S8—Ag2—O37	89.8(2)	S7—Ag40—S8	130.28(13)
S10—Ag2—S8	168.70(15)	O37—Ag40—S7	142.7(2)
S10—Ag2—O37	101.4(3)	O37—Ag40—S8	86.7(2)
S10—Ag3—S14	136.18(16)	O37—Mo1—O44	76.6(4)
S10—Ag3—O34	112.7(3)	O38—Mo1—O37	73.0(3)
O34—Ag3—S14	98.8(3)	O38—Mo1—O44	72.3(3)
S22—Ag4—S14	135.88(15)	O45—Mo1—O37	90.8(4)
O35—Ag4—S14	77.5(2)	O45—Mo1—O38	95.6(4)
O35—Ag4—S22	145.7(3)	O45—Mo1—O44	164.5(4)
S22—Ag5—S25^{i}	112.44(15)	O46—Mo1—O37	83.9(4)
O25—Ag5—S22	164.3(3)	O46—Mo1—O38	149.6(4)
O25—Ag5—S25^{i}	83.2(3)	O46—Mo1—O44	83.5(4)
O35—Ag6—O28	115.9(3)	O46—Mo1—O45	104.4(5)
O53—Ag6—O28	70.4(4)	O46—Mo1—O47	103.3(4)
O53—Ag6—O35	78.7(3)	O47—Mo1—O37	163.0(4)
O47^{i}—Ag7—O52	83.9(4)	O47—Mo1—O38	94.4(4)
S14—Ag8—S17	151.05(13)	O47—Mo1—O44	88.8(4)
O28—Ag8—S14	106.1(3)	O47—Mo1—O45	101.9(5)
O28—Ag8—S17	93.2(3)	O35—Mo2—O37	104.4(4)
O53—Ag8—S14	122.2(2)	O35—Mo2—O38	91.3(4)
O53—Ag8—S17	84.4(2)	O35—Mo2—O39	95.6(4)
O53—Ag8—O28	69.7(4)	O35—Mo2—O41	160.3(4)
S23—Ag9—S12^{i}	143.01(15)	O36—Mo2—O35	103.3(5)
O27—Ag9—S12^{i}	97.6(3)	O36—Mo2—O37	97.7(4)
O27—Ag9—S23	105.8(3)	O36—Mo2—O38	165.1(4)
S23—Ag10—S25^{i}	148.03(15)	O36—Mo2—O39	96.9(4)
S23—Ag10—O42^{i}	82.3(2)	O36—Mo2—O41	93.3(4)
S25^{i}—Ag10—O42^{i}	126.9(2)	O37—Mo2—O38	75.7(4)
O25—Ag10—S23	116.8(3)	O37—Mo2—O39	151.7(4)
O25—Ag10—S25^{i}	84.4(3)	O37—Mo2—O41	83.3(4)
O25—Ag10—O42^{i}	80.3(4)	O38—Mo2—O41	72.9(3)
S13^{i}—Ag11—S17	132.18(12)	O39—Mo2—O38	84.2(4)
S13^{i}—Ag11—O28	129.2(3)	O39—Mo2—O41	71.7(3)
O28—Ag11—S17	90.0(3)	O26—Mo3—O25	108.0(6)
S13^{i}—Ag12—S19	158.78(14)	O26—Mo3—O27	110.8(6)
S13^{i}—Ag13—S11^{i}	150.32(14)	O26—Mo3—O28	107.9(5)
O33—Ag13—S11^{i}	98.0(5)	O27—Mo3—O25	109.1(5)
O33—Ag13—S13^{i}	99.3(5)	O27—Mo3—O28	110.3(5)
O33—Ag13—O57	155.5(5)	O28—Mo3—O25	110.8(5)

O57—Ag13—S11^{i}	88.3(2)	O39—Mo4—O51	80.4(4)
O57—Ag13—S13^{i}	85.8(2)	O39—Mo4—O56	73.3(4)
S22^{i}—Ag14—S11^{i}	157.59(15)	O53—Mo4—O39	91.7(4)
O50—Ag14—S11^{i}	108.8(3)	O53—Mo4—O51	91.9(4)
O50—Ag14—S22^{i}	93.4(3)	O53—Mo4—O56	161.6(4)
S21—Ag15—O23	127.7(6)	O53—Mo4—O58	102.4(5)
O57—Ag15—S21	145.1(3)	O56—Mo4—O51	75.5(3)
O57—Ag15—O23	87.1(6)	O58—Mo4—O39	153.8(4)
S25—Ag16—S21	167.52(18)	O58—Mo4—O51	77.2(4)
S21—Ag17—S4	170.55(16)	O58—Mo4—O56	88.0(4)
S25—Ag18—S24	157.53(15)	O60—Mo4—O39	98.8(4)
O55—Ag18—S24	97.5(3)	O60—Mo4—O51	164.6(4)
O55—Ag18—S25	103.2(3)	O60—Mo4—O53	103.5(4)
S4—Ag19—S24	150.43(15)	O60—Mo4—O56	89.5(4)
S4—Ag19—O19	96.0(4)	O60—Mo4—O58	99.2(4)
S24—Ag19—O19	102.1(4)	O48—Mo5—O49	96.2(4)
O17—Ag19—S4	92.6(3)	O48—Mo5—O51	95.9(4)
O17—Ag19—S24	99.7(4)	O48—Mo5—O56	165.0(4)
O17—Ag19—O19	116.7(6)	O48—Mo5—O59	103.2(4)
S24—Ag20—S23^{i}	162.41(15)	O49—Mo5—O51	83.9(4)
O43—Ag20—S23^{i}	82.0(2)	O49—Mo5—O56	72.6(3)
O43—Ag20—S24	110.7(2)	O51—Mo5—O56	73.4(3)
S2—Ag21—S24	151.97(15)	O55—Mo5—O48	103.2(4)
S2—Ag21—O15	108.4(4)	O55—Mo5—O49	97.5(5)
S2—Ag21—O16	99.3(4)	O55—Mo5—O51	160.6(4)
S24—Ag21—O15	96.7(4)	O55—Mo5—O56	88.4(4)
S24—Ag21—O16	93.9(4)	O55—Mo5—O59	96.0(5)
O15—Ag21—O16	87.8(4)	O59—Mo5—O49	153.1(4)
S7—Ag22—S23^{i}	142.03(14)	O59—Mo5—O51	75.8(4)
O31—Ag22—S7	117.5(4)	O59—Mo5—O56	84.7(4)
O31—Ag22—S23^{i}	99.6(4)	O39—Mo6—O49	76.0(3)
S2—Ag23—S7	145.14(15)	O40—Mo6—O39	90.6(4)
O43—Ag23—S2	122.1(3)	O40—Mo6—O41	97.0(4)
O43—Ag23—S7	91.9(3)	O40—Mo6—O49	165.9(4)
S12—Ag24—S7	151.47(17)	O40—Mo6—O56	104.5(4)
S13—Ag25—S12	116.86(13)	O41—Mo6—O39	74.9(4)
S13—Ag25—O27^{i}	106.0(3)	O41—Mo6—O49	75.3(4)
O27^{i}—Ag25—S12	92.9(3)	O41—Mo6—O56	142.2(4)
O47—Ag25—S12	88.7(3)	O54—Mo6—O39	165.7(4)
O47—Ag25—S13	148.1(3)	O54—Mo6—O40	103.3(5)
O47—Ag25—O27^{i}	90.4(4)	O54—Mo6—O41	106.2(4)
S12—Ag26—S11	158.28(15)	O54—Mo6—O49	90.4(4)
S12—Ag26—O46	89.1(3)	O54—Mo6—O56	98.8(5)

O46—Ag26—S11	89.3(3)	O56—Mo6—O39	74.2(4)
S2—Ag27—O18	96.8(4)	O56—Mo6—O49	76.6(4)
S20—Ag27—S2	156.46(15)	O38—Mo7—O41	75.6(3)
S20—Ag27—O18	102.7(4)	O42—Mo7—O38	96.3(4)
O1—Ag27—S2	93.6(5)	O42—Mo7—O41	164.9(4)
O1—Ag27—S20	101.7(5)	O42—Mo7—O43	102.5(5)
O1—Ag27—O18	82.7(4)	O42—Mo7—O44	102.3(4)
S8—Ag28—S16	147.15(13)	O42—Mo7—O49	92.8(4)
S8—Ag28—O7	111.7(3)	O43—Mo7—O38	161.3(4)
O7—Ag28—S16	92.2(3)	O43—Mo7—O41	86.0(4)
S16—Ag29—S20	172.29(15)	O43—Mo7—O44	98.5(5)
S16—Ag29—O40	103.1(3)	O43—Mo7—O49	95.8(4)
S20—Ag29—O40	77.5(3)	O44—Mo7—O38	77.9(4)
S16—Ag30—S17	163.33(14)	O44—Mo7—O41	88.6(4)
S16—Ag30—O60	106.5(2)	O44—Mo7—O49	156.4(4)
S17—Ag30—O60	83.8(2)	O49—Mo7—O38	82.6(4)
S14—Ag31—S16	163.76(14)	O49—Mo7—O41	73.8(3)
S14—Ag31—O36	102.8(3)	O50—Mo8—O51	94.8(4)
S16—Ag31—O36	87.5(3)	O50—Mo8—O52	102.8(5)
S17—Ag32—S19	153.50(12)	O50—Mo8—O57	104.4(5)
S17—Ag32—O5	106.2(3)	O50—Mo8—O58	165.2(4)
S19—Ag32—O5	97.3(3)	O50—Mo8—O59	91.5(4)
S19—Ag33—S4	130.61(13)	O51—Mo8—O58	73.8(3)
S19—Ag33—O58	73.6(2)	O51—Mo8—O59	72.7(4)
O58—Ag33—S4	150.7(2)	O52—Mo8—O51	94.0(4)
O59—Ag33—S4	94.9(2)	O52—Mo8—O57	102.2(4)
O59—Ag33—S19	134.2(2)	O52—Mo8—O58	87.6(4)
O59—Ag33—O58	65.5(3)	O52—Mo8—O59	161.4(4)
S20—Ag34—S19	153.41(15)	O57—Mo8—O51	151.3(4)
O60—Ag34—S19	97.2(2)	O57—Mo8—O58	83.3(4)
O60—Ag34—S20	103.7(2)	O57—Mo8—O59	85.5(4)
S20—Ag35—S4	149.14(14)	O5—Mo8—O58	76.4(4)
Symmetry code: (i) −*x*+1, −*y*+1, −*z*+1.			
**SD/Ag80b**			
Ag1—Ag15^{i}	3.073 (3)	Ag24—O10^{i}	2.293 (5)
Ag1—Ag36	3.348 (12)	Ag24—S3	2.388 (2)
Ag1—O51^{i}	2.531 (17)	Ag24—S9	2.591 (2)
Ag1—S10	2.475 (6)	Ag25—Ag26	2.9429 (10)
Ag1—S17^{i}	2.498 (4)	Ag25—Ag39	3.3001 (11)
Ag2—Ag3	2.992 (4)	Ag25—O22	2.511 (6)
Ag2—Ag14	3.3489 (13)	Ag25—S3	2.420 (2)
Ag2—O19^{i}	2.394 (6)	Ag25—S19	2.416 (2)
Ag2—S3^{i}	2.353 (2)	Ag26—O1^{i}	2.395 (6)

Ag2—S16	2.458 (2)	Ag26—O40	2.542 (7)
Ag3—Ag15	3.365 (5)	Ag26—S4	2.437 (3)
Ag3—Ag38^{i}	3.079 (5)	Ag26—S19	2.631 (2)
Ag3—O55	2.364 (11)	Ag27—Ag28	3.0005 (10)
Ag3—S2^{i}	2.466 (5)	Ag27—Ag32	3.3522 (11)
Ag3—S16	2.534 (5)	Ag27—O24	2.503 (6)
Ag4—Ag19	2.9589 (15)	Ag27—S13	2.498 (3)
Ag4—O15^{i}	2.465 (6)	Ag27—S19	2.469 (3)
Ag4—S6	2.435 (3)	Ag28—Ag29	3.2478 (8)
Ag4—S17	2.410 (3)	Ag28—Ag31	3.2871 (11)
Ag5—Ag8	3.2525 (18)	Ag28—O3^{i}	2.322 (5)
Ag5—Ag20	3.291 (2)	Ag28—O29^{i}	2.511 (6)
Ag5—O53	2.533 (15)	Ag28—S13	2.467 (2)
Ag5—O57	2.55 (2)	Ag28—S14	2.595 (2)
Ag5—S5	2.446 (3)	Ag29—Ag30	3.0184 (11)
Ag5—S7	2.556 (4)	Ag29—O29^{i}	2.399 (6)
Ag6—Ag8	2.9787 (14)	Ag29—S14	2.519 (2)
Ag6—Ag9	2.9542 (12)	Ag29—S15	2.505 (2)
Ag6—Ag34^{i}	3.1910 (18)	Ag30—O4^{i}	2.558 (5)
Ag6—O14^{i}	2.543 (6)	Ag30—O42	2.593 (8)
Ag6—S7	2.413 (3)	Ag30—S14	2.436 (2)
Ag6—S8	2.499 (3)	Ag30—S18	2.457 (2)
Ag7—Ag27	2.971 (3)	Ag31—Ag9^{i}	3.2882 (11)
Ag7—O56	2.401 (13)	Ag31—Ag32	3.3538 (11)
Ag7—S14	2.551 (4)	Ag31—O25	2.521 (6)
Ag7—S19	2.531 (3)	Ag31—O30^{i}	2.478 (6)
Ag8—Ag9	2.9208 (11)	Ag31—S8^{i}	2.682 (3)
Ag8—Ag23	3.1131 (11)	Ag31—S13	2.486 (3)
Ag8—S7	2.445 (3)	Ag32—Ag33	2.8966 (12)
Ag8—S9	2.444 (2)	Ag32—Ag37	3.3730 (10)
Ag9—Ag11	3.2479 (9)	Ag32—O26	2.549 (5)
Ag9—Ag31^{i}	3.2882 (11)	Ag32—O34	2.593 (9)
Ag9—O25^{i}	2.497 (6)	Ag32—S12	2.493 (2)
Ag9—O30	2.437 (6)	Ag32—S13	2.473 (2)
Ag9—S8	2.542 (3)	Ag33—Ag35	3.0603 (10)
Ag9—S9	2.475 (3)	Ag33—S8^{i}	2.427 (2)
Ag10—Ag11	2.7210 (9)	Ag33—S12	2.445 (2)
Ag10—Ag12^{i}	2.8771 (8)	Ag34—Ag6^{i}	3.1910 (18)
Ag10—Ag13	2.8634 (8)	Ag34—Ag35	3.2373 (13)
Ag10—Ag13^{i}	2.9019 (9)	Ag34—O13	2.447 (6)
Ag10—Ag14^{i}	2.7005 (8)	Ag34—S7^{i}	2.414 (3)
Ag10—Ag24	3.0717 (10)	Ag34—S11	2.458 (3)
Ag10—O2^{i}	2.333 (6)	Ag35—Ag36	2.9014 (12)

Ag10—O22	2.322 (5)	Ag35—O14	2.462 (5)
Ag11—Ag12	2.6807 (10)	Ag35—O39	2.593 (7)
Ag11—Ag13	2.9166 (8)	Ag35—S11	2.509 (3)
Ag11—Ag14^{i}	2.9064 (9)	Ag35—S12	2.483 (3)
Ag11—Ag24	3.0568 (10)	Ag36—Ag37	3.0682 (14)
Ag11—O10^{i}	2.420 (6)	Ag36—S10	2.428 (3)
Ag11—O25^{i}	2.364 (5)	Ag36—S11	2.447 (3)
Ag11—O30	2.411 (5)	Ag37—Ag38	3.0675 (10)
Ag12—Ag10^{i}	2.8770 (8)	Ag37—O21	2.446 (5)
Ag12—Ag13^{i}	2.8133 (9)	Ag37—O26	2.576 (5)
Ag12—Ag13	2.8866 (8)	Ag37—S10	2.606 (2)
Ag12—Ag14^{i}	2.6977 (8)	Ag37—S12	2.554 (2)
Ag12—O3	2.294 (6)	Ag38—Ag3^{i}	3.079 (5)
Ag12—O23^{i}	2.316 (6)	Ag38—Ag39	3.0376 (12)
Ag13—Ag10^{i}	2.9020 (9)	Ag38—S2	2.413 (3)
Ag13—Ag12^{i}	2.8133 (9)	Ag38—S10	2.418 (3)
Ag13—Ag14	2.8950 (8)	Ag39—O24	2.206 (6)
Ag13—O5^{i}	2.375 (5)	Ag39—O35	2.524 (8)
Ag13—O18^{i}	2.338 (5)	Ag39—S2	2.393 (3)
Ag14—Ag10^{i}	2.7006 (8)	Ag40—O47	2.473 (9)
Ag14—Ag11^{i}	2.9063 (9)	Ag40—S6	2.449 (3)
Ag14—Ag12^{i}	2.6976 (8)	Ag40—S11^{i}	2.490 (3)
Ag14—Ag16	3.3529 (8)	Mo1—O27	1.729 (6)
Ag14—O6^{i}	2.405 (6)	Mo1—O28	1.782 (6)
Ag14—O19^{i}	2.362 (5)	Mo1—O29	1.748 (7)
Ag14—O28^{i}	2.326 (5)	Mo1—O30	1.795 (5)
Ag15—Ag1^{i}	3.073 (3)	Mo2—O1	1.762 (6)
Ag15—Ag16	3.1142 (9)	Mo2—O2	1.765 (5)
Ag15—Ag17	2.9300 (11)	Mo2—O3	1.777 (6)
Ag15—O20^{i}	2.578 (5)	Mo2—O4	2.189 (5)
Ag15—S16	2.407 (2)	Mo2—O5	2.091 (5)
Ag15—S17	2.445 (3)	Mo2—O9	2.185 (6)
Ag16—Ag17	3.0644 (10)	Mo3—O5	2.209 (5)
Ag16—O6^{i}	2.465 (5)	Mo3—O9	1.845 (6)
Ag16—O28^{i}	2.446 (6)	Mo3—O10	1.750 (5)
Ag16—S15	2.532 (2)	Mo3—O11	2.307 (5)
Ag16—S16	2.504 (2)	Mo3—O12	1.724 (6)
Ag17—Ag18	3.2553 (11)	Mo3—O16	1.949 (6)
Ag17—O7^{i}	2.552 (5)	Mo4—O4	1.812 (6)
Ag17—S15	2.485 (2)	Mo4—O5	2.173 (5)
Ag17—S17	2.460 (2)	Mo4—O6	1.744 (6)
Ag18—Ag19	3.2465 (11)	Mo4—O7	1.759 (5)
Ag18—Ag29	2.9896 (10)	Mo4—O8	1.991 (5)

Ag18—Ag30	2.9636 (9)	Mo4—O11	2.236 (6)
Ag18—O43	2.589 (7)	Mo5—O8	2.171 (6)
Ag18—S15	2.472 (3)	Mo5—O11	1.929 (6)
Ag18—S18	2.449 (3)	Mo5—O13	1.741 (6)
Ag19—Ag20	2.9884 (13)	Mo5—O15	1.723 (5)
Ag19—Ag21	3.3134 (10)	Mo5—O16	2.164 (5)
Ag19—O7^{i}	2.414 (6)	Mo5—O17	1.939 (6)
Ag19—S6	2.488 (2)	Mo6—O8	1.944 (6)
Ag19—S18	2.487 (3)	Mo6—O17	2.343 (6)
Ag20—Ag21	3.0894 (13)	Mo6—O18	2.210 (5)
Ag20—Ag40	3.3284 (14)	Mo6—O19	1.745 (6)
Ag20—S5	2.424 (3)	Mo6—O20	1.727 (5)
Ag20—S6	2.459 (2)	Mo6—O21	1.838 (6)
Ag21—Ag22	3.0376 (10)	Mo7—O18	2.018 (5)
Ag21—Ag30	3.2419 (11)	Mo7—O21	2.199 (5)
Ag21—O9^{i}	2.461 (5)	Mo7—O22	1.752 (6)
Ag21—S5	2.659 (3)	Mo7—O23	1.760 (5)
Ag21—S18	2.551 (2)	Mo7—O24	1.799 (6)
Ag22—Ag23	3.1686 (10)	Mo7—O26	2.223 (6)
Ag22—O9^{i}	2.579 (6)	Mo8—O14	1.747 (6)
Ag22—S4	2.390 (2)	Mo8—O16	1.976 (6)
Ag22—S5	2.425 (3)	Mo8—O17	2.199 (5)
Ag23—Ag24	3.0223 (12)	Mo8—O18	2.238 (5)
Ag23—O32	2.59 (3)	Mo8—O25	1.757 (5)
Ag23—S4	2.425 (3)	Mo8—O26	1.810 (6)
Ag23—S9	2.508 (3)		
S10—Ag1—O51^{i}	100.9(4)	O21—Ag37—S10	94.08(13)
S10—Ag1—S17^{i}	157.3(5)	O21—Ag37—S12	138.56(13)
S17^{i}—Ag1—O51^{i}	94.7(5)	O26—Ag37—S10	144.69(13)
O19^{i}—Ag2—S16	86.15(14)	S12—Ag37—O26	75.53(13)
S3^{i}—Ag2—O19^{i}	120.94(14)	S12—Ag37—S10	126.41(8)
S3^{i}—Ag2—S16	152.40(9)	S2—Ag38—S10	169.71(9)
O55—Ag3—S2^{i}	104.6(3)	O24—Ag39—O35	84.5(3)
O55—Ag3—S16	100.2(3)	O24—Ag39—S2	153.77(16)
S21—Ag3—S16	138.52(17)	S2—Ag39—O35	121.6(2)
S6—Ag4—O15^{i}	80.25(17)	S6—Ag40—O47	101.6(3)
S17—Ag4—O15^{i}	95.6(2)	S6—Ag40—S11^{i}	157.21(9)
S17—Ag4—S6	168.27(10)	O27—Mo1—O28	108.5(3)
O57—Ag5—O53	69.6(5)	O27—Mo1—O29	110.1(3)
O57—Ag5—S7	100.5(4)	O27—Mo1—O30	108.7(3)
S5—Ag5—O53	112.2(3)	O28—Mo1—O30	110.5(3)
S5—Ag5—O57	100.6(4)	O29—Mo1—O28	110.1(3)
S5—Ag5—S7	155.49(13)	O29—Mo1—O30	108.8(3)

S7—Ag5—O53	86.8(3)	O1—Mo2—O2	104.9(3)
S7—Ag6—O14^{i}	105.09(16)	O1—Mo2—O3	103.4(3)
S7—Ag6—S8	163.70(10)	O1—Mo2—O4	84.3(2)
S8—Ag6—O14^{i}	84.15(15)	O1—Mo2—O5	149.4(2)
O56—Ag7—S14	93.5(3)	O1—Mo2—O9	84.0(3)
O56—Ag7—S19	116.4(3)	O2—Mo2—O3	102.2(3)
S19—Ag7—S14	141.0(2)	O2—Mo2—O4	163.6(2)
S9—Ag8—S7	164.31(11)	O2—Mo2—O5	94.6(2)
O25^{i}—Ag9—S8	83.09(13)	O2—Mo2—O9	90.3(2)
O30—Ag9—O25^{i}	70.63(18)	O3—Mo2—O4	88.6(2)
O30—Ag9—S8	94.55(16)	O3—Mo2—O5	95.1(2)
O30—Ag9—S9	102.29(16)	O3—Mo2—O9	163.1(2)
S9—Ag9—O25^{i}	119.70(14)	O5—Mo2—O4	71.87(19)
S9—Ag9—S8	155.08(9)	O5—Mo2—O9	72.3(2)
O22—Ag10—O2^{i}	78.08(19)	O9—Mo2—O4	76.9(2)
O25^{i}—Ag11—O10^{i}	80.0(2)	O5—Mo3—O11	73.13(18)
O25^{1}—Ag11—O30	73.37(18)	O9—Mo3—O5	76.5(2)
O30—Ag11—O10^{i}	119.9(2)	O9—Mo3—O11	83.5(2)
O3—Ag12—O23^{i}	84.4(2)	O9—Mo3—O16	152.4(2)
O18^{i}—Ag13—O5^{i}	98.64(18)	O10—Mo3—O5	91.9(2)
O19^{i}—Ag14—O6^{i}	80.42(19)	O10—Mo3—O9	104.6(3)
O28^{i}—Ag14—O6^{i}	82.6(2)	O10—Mo3—O11	161.0(2)
O28^{i}—Ag14—O19^{i}	99.08(19)	O10—Mo3—O16	95.4(2)
S16—Ag15—O20^{i}	97.01(14)	O12—Mo3—O5	165.0(2)
S16—Ag15—S17	162.34(8)	O12—Mo3—O9	97.4(3)
S17—Ag15—O20^{i}	93.00(15)	O12—Mo3—O10	102.9(3)
O6^{i}—Ag16—S15	82.66(14)	O12—Mo3—O11	92.8(2)
O6^{i}—Ag16—S16	133.86(14)	O12—Mo3—O16	96.3(3)
O28^{i}—Ag16—O6^{i}	78.96(18)	O16—Mo3—O5	84.3(2)
O28^{i}—Ag16—S15	114.28(16)	O16—Mo3—O11	72.0(2)
O28^{i}—Ag16—S16	91.13(16)	O4—Mo4—O5	77.6(2)
S16—Ag16—S15	140.43(7)	O4—Mo4—O8	155.4(2)
S15—Ag17—O7^{i}	78.62(13)	O4—Mo4—O11	88.0(2)
S17—Ag17—O7^{i}	106.43(13)	O5—Mo4—O11	75.23(19)
S17—Ag17—S15	166.35(9)	O6—Mo4—O5	94.8(2)
S15—Ag18—O43	108.0(2)	O6—Mo4—O7	103.8(2)
S18—Ag18—O43	97.6(2)	O6—Mo4—O8	93.0(2)
S18—Ag18—S15	149.72(8)	O6—Mo4—O11	163.7(2)
O7^{i}—Ag19—S6	123.82(15)	O7—Mo4—O4	98.6(2)
O7^{i}—Ag19—S18	89.83(14)	O7—Mo4—O5	161.5(2)
S18—Ag19—S6	141.46(9)	O7—Mo4—O8	96.0(2)
S5—Ag20—S6	151.64(9)	O7—Mo4—O11	86.6(2)
O9^{i}—Ag21—S5	91.15(14)	O8—Mo4—O5	82.34(19)

O9^{i}—Ag21—S18	140.53(15)	O8—Mo4—O11	73.1(2)
S18—Ag21—S5	127.60(8)	O11—Mo5—O8	75.8(2)
S4—Ag22—O9^{i}	97.99(13)	O11—Mo5—O16	75.7(2)
S4—Ag22—S5	168.07(10)	O11—Mo5—O17	143.1(2)
S5—Ag22—O9^{i}	93.90(13)	O13—Mo5—O8	166.2(2)
S4—Ag23—O32	98.1(5)	O13—Mo5—O11	97.2(3)
S4—Ag23—S9	145.77(9)	O13—Mo5—O16	90.0(2)
S9—Ag23—O32	99.5(6)	O13—Mo5—O17	105.2(3)
O10^{i}—Ag24—S3	152.11(15)	O15—Mo5—O8	90.7(2)
O10^{i}—Ag24—S9	78.34(15)	O15—Mo5—O11	105.8(3)
S3—Ag24—S9	129.14(8)	O15—Mo5—O13	102.7(3)
S3—Ag25—O22	84.59(14)	O15—Mo5—O16	166.8(3)
S19—Ag25—S3	158.13(9)	O15—Mo5—O17	97.6(3)
O1^{i}—Ag26—O40	89.4(2)	O16—Mo5—O8	76.81(19)
O1^{i}—Ag26—S4	124.74(15)	O17—Mo5—O8	75.7(2)
O1^{i}—Ag26—S19	82.86(15)	O17—Mo5—O16	75.3(2)
O40—Ag26—S19	95.81(19)	O8—Mo6—O17	71.6(2)
S4—Ag26—O40	103.2(2)	O8—Mo6—O18	83.0(2)
S4—Ag26—S19	145.96(8)	O18—Mo6—O17	73.15(18)
S13—Ag27—O24	87.03(15)	O19—Mo6—O8	95.5(3)
S19—Ag27—O24	97.57(15)	O19—Mo6—O17	162.3(2)
S19—Ag27—S13	159.95(8)	O19—Mo6—O18	93.7(2)
O3^{i}—Ag28—O29^{i}	86.8(2)	O19—Mo6—O21	103.9(3)
O3^{i}—Ag28—S13	144.03(14)	O20—Mo6—O8	98.0(2)
O3^{i}—Ag28—S14	90.37(15)	O20—Mo6—O17	90.7(2)
O29^{i}—Ag28—S14	90.75(15)	O20—Mo6—O18	162.7(2)
S13—Ag28—O29^{i}	112.24(16)	O20—Mo6—O19	103.3(3)
S13—Ag28—S14	118.29(8)	O20—Mo6—O21	97.3(2)
O29^{i}—Ag29—S14	95.29(16)	O21—Mo6—O8	151.7(2)
O29^{i}—Ag29—S15	107.51(15)	O21—Mo6—O17	84.5(2)
S15—Ag29—S14	147.36(8)	O21—Mo6—O18	75.5(2)
O4^{i}—Ag30—O42	128.5(2)	O18—Mo7—O21	72.5(2)
S14—Ag30—O4^{i}	100.14(14)	O18—Mo7—O26	72.7(2)
S14—Ag30—O42	97.2(2)	O21—Mo7—O26	75.0(2)
S14—Ag30—S18	157.04(8)	O22—Mo7—O18	96.5(2)
S18—Ag30—O4^{i}	81.23(13)	O22—Mo7—O21	91.7(2)
S18—Ag30—O42	99.8(2)	O22—Mo7—O23	103.5(3)
O25—Ag31—S8^{i}	79.87(14)	O22—Mo7—O24	103.3(3)
O30^{i}—Ag31—O25	69.59(17)	O22—Mo7—O26	164.8(2)
O30^{i}—Ag31—S8^{i}	90.23(15)	O23—Mo7—O18	95.1(2)
O30^{i}—Ag31—S13	136.90(15)	O23—Mo7—O21	161.5(3)
S13—Ag31—O25	137.70(14)	O23—Mo7—O24	102.6(3)
S13—Ag31—S8^{i}	122.21(8)	O23—Mo7—O26	88.4(2)

O26—Ag32—O34	139.9(2)	O24—Mo7—O18	149.4(2)
S12—Ag32—O26	77.09(13)	O24—Mo7—O21	83.7(2)
S12—Ag32—O34	98.5(2)	O24—Mo7—O26	82.9(2)
S13—Ag32—O26	101.90(13)	O14—Mo8—O16	98.3(3)
S13—Ag32—O34	96.97(19)	O14—Mo8—O17	89.5(2)
S13—Ag32—S12	156.31(9)	O14—Mo8—O18	164.6(2)
$S8^{i}$—Ag33—S12	155.43(8)	O14—Mo8—O25	103.2(3)
O13—Ag34—S11	77.76(15)	O14—Mo8—O26	100.4(3)
$S7^{i}$—Ag34—O13	106.41(15)	O16—Mo8—O17	73.8(2)
$S7^{i}$—Ag34—S11	171.77(11)	O16—Mo8—O18	80.8(2)
O14—Ag35—O39	90.9(2)	O17—Mo8—O18	75.43(19)
O14—Ag35—S11	102.84(15)	O25—Mo8—O16	91.5(2)
O14—Ag35—S12	95.54(16)	O25—Mo8—O17	161.9(2)
S11—Ag35—O39	93.91(19)	O25—Mo8—O18	92.2(2)
S12—Ag35—O39	108.13(19)	O25—Mo8—O26	102.0(3)
S12—Ag35—S11	151.03(8)	O26—Mo8—O16	153.7(2)
S10—Ag36—S11	153.28(9)	O26—Mo8—O17	88.0(2)
O21—Ag37—O26	64.77(17)	O26—Mo8—O18	76.2(2)
Symmetry code: (i) $-x$, $-y+1$, $-z$.			

Table S5: Bond valence sum (BVS) calculations for the valences of Mo in SD/Ag80a and SD/Ag80b.

SD/Ag80a								
Atoms	Mo1	Mo2	Mo3	Mo4	Mo5	Mo6	Mo7	Mo8
Valence	5.818	5.821	5.871	5.910	5.829	5.938	5.862	5.795
**SD/Ag80b**								
Atoms	Mo1	Mo2	Mo3	Mo4	Mo5	Mo6	Mo7	Mo8
Valence	5.848	5.842	5.972	5.911	5.963	5.975	5.911	5.958

Table S6: The structures and coordination modes of molybdates toward Ag atoms found in silver clusters in the literature and this work.

Silver clusters	Molybdate	Number of Ag	Ref
$[Ag_{62}(S^{t}Bu)_{40}(Mo_{20}O_{66})(Mo_6O_{19})_3(CH_3CN)_2]\cdot(CF_3SO_3)_4$	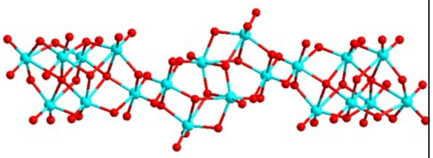 $[Mo_{20}O_{66}]^{12-}$	44	7
$[Mo_6O_{22}@Ag_{46}(^{t}BuC_6H_4S)_{32}(dppm)_4(CH_3CN)_8]\cdot 6CF_3SO_3$	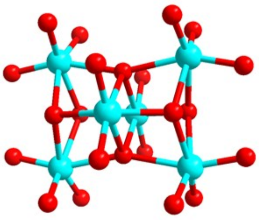 $[Mo_6O_{22}]^{8-}$	28	8
$Mo_6O_{22}@Ag_{58}S_2(SC_6H_4{}^{t}Bu)_{36}(CF_3COO)_{10}(H_2O)_8$	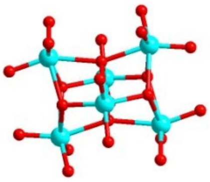 $[Mo_6O_{22}]^{8-}$	24	9
$Mo_7O_{24}@Ag_{41}(^{i}PrS)_{19}(p\text{-}TOS)_{16}(CH_3OH)_4\cdot 4CH_3OH$	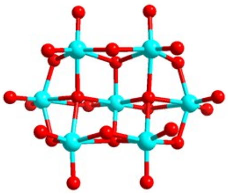 $[Mo_7O_{24}]^{6-}$	26	10
$(^{n}Bu_4N)_{1.5}[Mo_5O_{18}@Ag_{36}(iPrS)_{18}(p\text{-}TOS)_{13.5}(CH_3CN)\cdot 1.5CH_3CN$	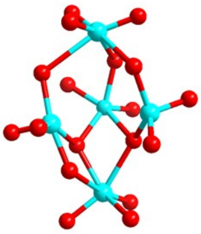 $[Mo_5O_{18}]^{6-}$	25	10
$(Mo_6O_{22})_2@Ag_{76}(MeOC_6H_4S)_{28}(dppm)_8(MoO_4)_{16}(H_2O)_8\cdot 8CH_3OH\cdot 4CH_3CN$	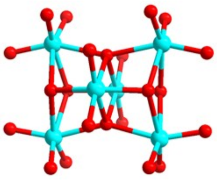	26	11

	$[Mo_6O_{22}]^{8-}$		
$[(\alpha\text{-}Mo_5O_{18})@Ag_{38}(^{t}BuS)_{18}(PhCOO)_{14}\cdot 2CH_2Cl_2]$	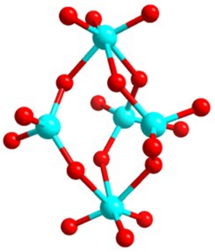<br>$[\alpha\text{-}Mo_5O_{18}]^{6-}$	27	12
$(\alpha\text{-}Mo_5O_{18})@Ag_{36}(^{i}PrS)_{18}(PhSO_3)_{12}(DMF)_6$	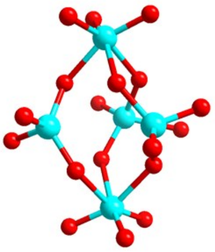<br>$[\alpha\text{-}Mo_5O_{18}]^{6-}$	18	1
$\{(^{n}Bu_4NH)[(\beta\text{-}Mo_5O_{18})@Ag_{36}(^{i}BuS)_{18}(PhSO_3)_{13}(CH_3OH)]\}_n$	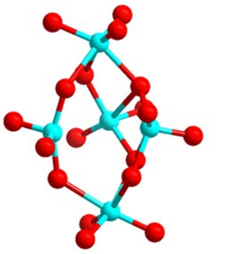<br>$[\beta\text{-}Mo_5O_{18}]^{6-}$	20	1
$[Mo_2O_8@Ag_{30}(^{i}BuS)_{15}(PhSO_3)_{11}(CH_3OH)_2(H_2O)\cdot H_2O]_2$	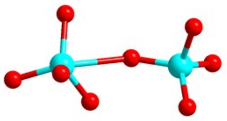<br>$[Mo_2O_8]^{4-}$	14	1
$\{[Mo_4O_{14}(SO_4)]_2@Ag_{73}S_4(PhSO_3)_{17}(^{i}BuS)_{30}(SO_4)_3(H_2O)_4\cdot 2H_2O\}$	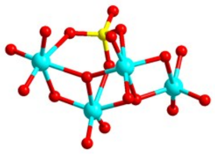<br>$[Mo_4O_{14}(SO_4)]^{6-}$	23	1
$Mo_6O_{22}@Ag_{40}(C{\equiv}C^{t}Bu)_{20}(CF_3COO)_{12}$	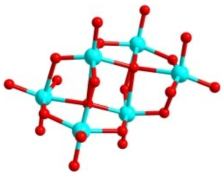<br>$[Mo_6O_{22}]^{8-}$	24	13

$[(Mo_6O_{22})_2@Ag_{60}(C{\equiv}C^tBu)_{38}](CF_3SO_3)_6$	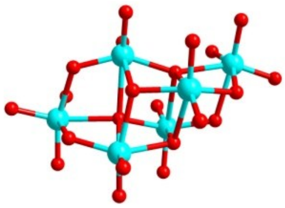 $[Mo_6O_{22}]^{8-}$	22	14
$[Ag_{10}@(Mo_7O_{26})_2@Ag_{70}(MoO_4)_2(CyhS)_{36}(CF_3SO_3)_{16}(DMF)_6]\cdot 2DMF\cdot 4^nPrOH$	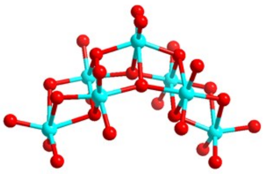 $Mo_7O_{26}^{10-}$	35	This work

Reference:

1. Z. Wang, H.-F. Su, X.-P. Wang, Q.-Q. Zhao, C.-H. Tung, D. Sun, L.-S. Zheng, *Chem-Eur J.*, 2018, **24**, 1640-1650.
2. *CrysAlisPro*, Rigaku OD, The Woodlands, TX, 2015.
3. L. Palatinus, G. Chapuis, *J. Appl. Crystallogr.*, 2007, **40**, 786-790.
4. G. M. Sheldrick, *Acta. Crystallogr. Sect. C.*, 2015, **71**, 3-8.
5. O. V. Dolomanov, L. J. Bourhis, R. J. Gildea, J. A. K. Howard, H. Puschmann, *J. Appl. Crystallogr.*, 2009, **42**, 339-341.
6. A. L. Spek, *Acta. Crystallogr. Sect. D.*, 2009, **65**, 148-155.
7. R.-W. Huang, Q.-Q. Xu, H.-L. Lu, X.-K. Guo, S.-Q. Zang, G.-G. Gao, M.-S. Tang, T. C. W. Mak, *Nanoscale*, 2015, **7**, 7151-7154.
8. X.-Y. Li, Z. Wang, H.-F. Su, S. Feng, M. Kurmoo, C.-H. Tung, D. Sun, L.-S. Zheng, *Nanoscale*, 2017, **9**, 3601-3608.
9. X.-Y. Li, Y.-Z. Tan, K. Yu, X.-P. Wang, Y.-Q. Zhao, D. Sun, L.-S. Zheng, *Chem-Asian J.*, 2015, **10**, 1295-1298.
10. Z. Wang, H.-F. Su, M. Kurmoo, C.-H. Tung, D. Sun, L.-S Zheng, *Nat. Commun.*, 2018, **9**, 2094.
11. J.-W. Liu, L. Feng, H.-F. Su, Z. Wang, Q.-Q. Zhao, X.-P. Wang, C.-H. Tung, D. Sun, L.-S. Zheng, *J. Am. Chem. Soc.*, 2018, **140**, 1600-1603.
12. Y.-M. Su, W. Liu, Z. Wang, S.-A. Wang, Y.-A. Li, F. Yu, Q.-Q. Zhao, X.-P.Wang, C.-H. Tung, D. Sun, *Chem-Eur J.*, 2018, **24**, 4967-4972.
13. G. G. Gao, P. S. Cheng, T. C. W. Mak, *J. Am. Chem. Soc.*, 2009, **131**, 18257-18259.
14. J. Qiao, Q. M. Wang, *Angew. Chem., Int. Ed.*, 2010, **49**, 1765-1767.
